# Accumulation of Large Lineage-Specific Repeats Coincides with Sequence Acceleration and Structural Rearrangement in *Plantago* Plastomes

**DOI:** 10.1093/gbe/evae177

**Published:** 2024-08-27

**Authors:** Jie Wang, Shenglong Kan, Jiali Kong, Liyun Nie, Weishu Fan, Yonglin Ren, Wayne Reeve, Jeffrey P Mower, Zhiqiang Wu

**Affiliations:** Shenzhen Branch, Guangdong Laboratory of Lingnan Modern Agriculture, Key Laboratory of Synthetic Biology, Ministry of Agriculture and Rural Affairs, Agricultural Genomics Institute at Shenzhen, Chinese Academy of Agricultural Sciences, Shenzhen 518120, China; School of Medical, Molecular and Forensic Sciences, Murdoch University, Perth, WA 6150, Australia; College of Environmental and Life Sciences, Murdoch University, Perth, WA 6150, Australia; Shenzhen Branch, Guangdong Laboratory of Lingnan Modern Agriculture, Key Laboratory of Synthetic Biology, Ministry of Agriculture and Rural Affairs, Agricultural Genomics Institute at Shenzhen, Chinese Academy of Agricultural Sciences, Shenzhen 518120, China; Marine College, Shandong University, Weihai 264209, China; Shenzhen Branch, Guangdong Laboratory of Lingnan Modern Agriculture, Key Laboratory of Synthetic Biology, Ministry of Agriculture and Rural Affairs, Agricultural Genomics Institute at Shenzhen, Chinese Academy of Agricultural Sciences, Shenzhen 518120, China; Shenzhen Branch, Guangdong Laboratory of Lingnan Modern Agriculture, Key Laboratory of Synthetic Biology, Ministry of Agriculture and Rural Affairs, Agricultural Genomics Institute at Shenzhen, Chinese Academy of Agricultural Sciences, Shenzhen 518120, China; School of Medical, Molecular and Forensic Sciences, Murdoch University, Perth, WA 6150, Australia; Institute of Genetics and Developmental Biology, Chinese Academy of Sciences, Beijing 100101, China; College of Environmental and Life Sciences, Murdoch University, Perth, WA 6150, Australia; School of Medical, Molecular and Forensic Sciences, Murdoch University, Perth, WA 6150, Australia; Center for Plant Science Innovation, University of Nebraska, Lincoln, NE 68588, USA; Department of Agronomy and Horticulture, University of Nebraska, Lincoln, NE 68583, USA; Shenzhen Branch, Guangdong Laboratory of Lingnan Modern Agriculture, Key Laboratory of Synthetic Biology, Ministry of Agriculture and Rural Affairs, Agricultural Genomics Institute at Shenzhen, Chinese Academy of Agricultural Sciences, Shenzhen 518120, China

**Keywords:** plastid, substitution rate, inversion, repetitive DNA, inverted repeats, *Plantago*

## Abstract

Repeats can mediate rearrangements and recombination in plant mitochondrial genomes and plastid genomes. While repeat accumulations are linked to heightened evolutionary rates and complex structures in specific lineages, debates persist regarding the extent of their influence on sequence and structural evolution. In this study, 75 *Plantago* plastomes were analyzed to investigate the relationships between repeats, nucleotide substitution rates, and structural variations. Extensive repeat accumulations were associated with significant rearrangements and inversions in the large inverted repeats (IRs), suggesting that repeats contribute to rearrangement hotspots. Repeats caused infrequent recombination that potentially led to substoichiometric shifting, supported by long-read sequencing. Repeats were implicated in elevating evolutionary rates by facilitating localized hypermutation, likely through DNA damage and repair processes. This study also observed a decrease in nucleotide substitution rates for loci translocating into IRs, supporting the role of biased gene conversion in maintaining lower substitution rates. Combined with known parallel changes in mitogenomes, it is proposed that potential dysfunction in nuclear-encoded genes associated with DNA replication, recombination, and repair may drive the evolution of *Plantago* organellar genomes. These findings contribute to understanding how repeats impact organellar evolution and stability, particularly in rapidly evolving plant lineages.

**Significance**

Repeats are known to be important in shaping the architecture of plant organellar genomes. The extent of their influence on sequence and structural evolution remains especially intriguing. This research revealed the roles of repeats in augmenting structural complexity and increasing evolutionary rates in *Plantago* plastomes, particularly the infrequent recombination uncommon in other plastomes, as well as the complicated effects that translocated loci suffered. The study contributed to a more comprehensive understanding of repeat-mediated and inverted repeat-expanded effects on plastome evolution, especially in fast-evolving clades.

## Introduction

The presence of repeats in plant mitochondrial genomes (mitogenomes), spanning from base pairs (bp) to several kilobases (kb), induces complex structural rearrangements through (il)legitimate recombination (Wynn and Christensen 2019; Wu et al. 2020a; Odahara et al. 2021; Lai et al. 2022; Zhang et al. 2023). While animal mitogenomes lack abundant repeats and exhibit fewer rearrangements, plant plastid genomes (plastomes) possess numerous repeats but generally fewer rearrangements compared with plant mitogenomes, potentially because most plastid repeats tend to be smaller. Various genomic isomers, resulting from recombination at repeats, can coexist within an individual and undergo shifts over evolutionary time, a phenomenon referred to as substoichiometric shifting (SSS). The scarcity of abundant repeats explains the relative absence of rearrangements in animal mitogenomes (Boore 1999). Conversely, plastomes possess numerous repeats of varying sizes; however, a single plastome generally exhibits fewer rearrangements than a mitogenome, with some exceptions in lineages such as *Carex*, *Selaginella*, and *Eleocharis* (Chumley et al. 2006; Sloan et al. 2012b; Kang et al. 2020; Lee et al. 2020; Xiang et al. 2022). In certain cupressophytes, SSS has been confirmed in plastomes, although further investigation is needed to understand the mechanisms underlying the abundance shift and the evolutionary implications on the species (Guo et al. 2014). A distinctive feature in typical land plant plastomes is the presence of inverted repeats (IRs), actively altering two interchangeable configurations in vivo to generate two equimolar major isomers. The two major isomers differ in their relative orientations of the large single-copy (LSC) and small single-copy (SSC) regions. Some studies assumed that intact IRs play a crucial role in maintaining plastome stability and preserving the quadripartite structure (Palmer and Thompson 1982; Wang and Lanfear 2019). However, recent research challenged this notion, noting observations in plant lineages with intact IRs but highly variable plastomes, such as *Selaginella*, *Pelargonium*, legumes, and gnetophytes; conversely, some plastomes from lineages lacking IRs could also exhibit fewer rearrangements (Mower and Vickrey 2018; Wang et al. 2022). The stabilization function of IRs was also challenged in recent research on photosynthetic euglenids (Maciszewski et al. 2022). Instead, a more recent idea is that various processes, shaping both structural and sequence plastome evolution, lead to IR loss, with repeat-mediated recombination being one appealing possibility (Blazier et al. 2016; Zhang et al. 2019; Wang et al. 2022, 2024).

Although IRs are generally conserved in most land plants, significant variations exist in independent lineages, including extreme contractions in *Pinus*, expansions in *Pelargonium* and *Plantago*, and loss in species within Papilionoideae, *Passiflora*, and cupressophytes (Wu et al. 2011; Guo et al. 2014; Weng et al. 2017; Mower and Vickrey 2018; Choi et al. 2019; Cauz Santos et al. 2020; Mower et al. 2021a; Maciszewski et al. 2022). Additionally, structural variations, such as large-scale inversions in IRs, are also observed (Wolf et al. 2010; Lee et al. 2020, 2023a; Wang et al. 2024). Atypical IR lineages tend to coincide with an increase in repeats, a high complexity of plastome structures, and elevated substitution rates (Weng et al. 2013; Schwarz et al. 2017; Lai et al. 2022; Zou et al. 2022). While abundant and varying-sized plastid repeats can exist in certain lineages, their abilities to mediate rearrangements largely differ (Chumley et al. 2006). In many cases, longer repeats may exhibit higher activities (Wynn and Christensen 2019; Wu et al. 2020a; Lee et al. 2023b). Moreover, if present, repeats can cause local rearrangements, leading to variations in nearby structures and sequences—a phenomenon referred to as localized hypermutation (Magee et al. 2010; Mower et al. 2021a). Beyond structural considerations, shifts in IR boundaries can include genes originally located in single-copy (SC) regions or exclude genes originally located in IR regions, thereby influencing their evolutionary rates (Maciszewski et al. 2022). Synonymous substitution rates (*d*_S_) are nearly four times slower in IRs than in SC regions, and *d*_S_ will decrease or increase when genes are translocated into or out of IRs, respectively (Perry and Wolfe 2002; Zhu et al. 2016; Park et al. 2018; Wang et al. 2022). However, certain genes from *Plantago*, *Pelargonium*, and *Silene* unexpectedly exhibit substantially rapid *d*_S_ when located in IRs (Sloan et al. 2014; Zhu et al. 2016; Weng et al. 2017; Mower et al. 2021a), raising questions regarding the ubiquity and extent to which these repeats shape plastome evolution, especially in lineages that are otherwise characterized by rapid evolution and high disorder.

*Plantago* (Plantaginaceae) encompasses approximately 250 species distributed globally across four subgenera, namely *Bougueria*, *Coronopus*, *Plantago*, and *Psyllium* (Hassemer et al. 2019; Mower et al. 2021a; Xie et al. 2023). Previously, atypical changes in *Plantago* plastomes were documented, revealing increased rearrangements, repeats, intron losses, indels, severe IR boundary expansions, and elevated substitution rates, setting them apart from the characteristics of most land plants (Zhu et al. 2016; Mower et al. 2021a). Initial comparisons between two *Plantago* species (*Plantago media* and *Plantago maritima*) were made (Zhu et al. 2016), and a subsequent broader sampling within the tribe Plantagineae further explored plastome-wide variants (Mower et al. 2021a). This investigation unveiled several crucial features, particularly the presence of two inversions exceeding 10 kb in the IRs of subgenera *Plantago* and *Coronopus*. Consequently, IR expansion occurred to varying degrees, leading to different numbers of original SSC genes translocating into the IR. The substantial and synchronous expansions of and inversions in the IRs among closely related subgenera in *Plantago* presented a valuable opportunity for a systematic exploration of the impact of highly variable IRs on shaping plastomes in both sequence and structure. Despite certain features being primarily elucidated in *Plantago*, uncertainties persist regarding the extent to which significant inversions within IRs influence plastome evolution and how repeats are linked to variations in evolutionary rates and structures.

In this study, by combining previously published and newly generated *Plantago* completed plastomes, a comprehensive sampling covering all four subgenera and 16 sections was carried out to compare whole-plastome-wide heterogeneities in sequence composition and structural organization, and the changes in translocated genes that migrated from SC to IR, aiming to (i) investigate the patterns of global variants across the genus, (ii) detect the influence of repeats on changes to nucleotide substitution and organization, and (iii) elucidate the impact of IRs on translocated genes.

## Results

### Clade-Specific Variants in Plastome Composition and Organization

**Fig. 1.**
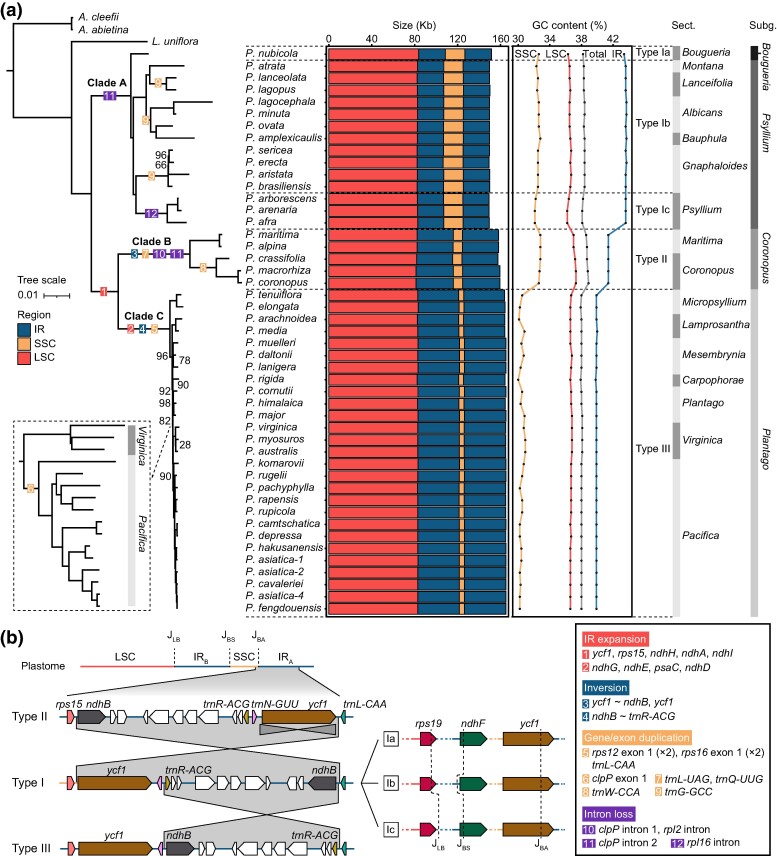
A phylogram of *Plantago* plastomes with IR boundary shifts, GC content changes, and inferred genomic reorganizations. a) The left panel displays the ML tree based on the concatenation of common CDSs. Bootstrap values below 100% are indicated at nodes. Genomic variants are denoted on branches, with details summarized in the box at the bottom right. The stacked histograms and line charts illustrate the sizes and GC contents of LSC, SSC, and IR for each species in the middle. Plastome types are labeled on the right. b) The schematic diagram illustrates inversions among different plastome types. The bars on the top represent LSC, IR, and SSC. The vertical dotted lines indicate junctions between IR and SC. The gray regions signify inversions among the three main types (I, II, and III). Gene locations to IR–SC junctions of three minor plastome types (a, b, and c) within type I are depicted in the middle.

The phylogenetic reconstruction based on coding sequences (CDSs) was consistent with that of previous analyses (Hassemer et al. 2019; Mower et al. 2021a), confirming the division of *Plantago* into four subgenera comprising 16 sections. The genus *Plantago* was further delineated into three primary evolutionary clades (Fig. 1a). *Plantago* plastomes exhibited significant size variation, approximately 16 kb, with *Plantago arborescens* (Clade A) having the smallest size at 149,298 bp and *P. major* (Clade C) having the largest size at 165,082 bp. The alterations in plastome size were predominantly attributed to the expansion of the IR into SSC in Clade B and Clade C (Fig. 1a; supplementary fig. S2a to d and supplementary table S2, Supplementary Material online). Moreover, from Clade A to Clade C, the GC content of both the IR and the SSC decreased by 4% and 3%, respectively. This phenomenon led to a robust negative correlation between GC content and IR size, substantiated by both standard linear models and phylogenetic generalized least squares (PGLS) analyses (supplementary fig. S2e to h and supplementary table S3, Supplementary Material online).

While the gene content remained generally consistent across clades (79 unique protein-coding genes [PCGs], 30 unique transfer RNA [tRNA] genes, and 4 unique ribosomal RNA genes), specific tRNA or protein-coding exon duplications unique to certain clades were identified (Fig. 1a). Intriguingly, a few introns were lost, including the first intron of *clpP* and the intron of *rpl2* in Clade B, the second intron of *clpP* in both Clade A and Clade B, and the *rpl16* intron in section *Psyllium*.

In Clade B and Clade C, expansions of the IR into SSC were evident. In Clade B, five original SSC-located PCGs, namely, *ycf1*, *rps15*, *ndhH*, *ndhA*, and *ndhI*, were translocated into the IR as part of the 10-kb IR expansion. In Clade C, nine PCGs, including the aforementioned five, as well as *ndhG*, *ndhE*, *psaC*, and *ndhD*, were translocated into the IR by the ∼14-kb IR expansion (Fig. 1a). Based on significant inversions within the IR and minor IR boundary shifts, the following three major plastome types were identified. Type I (Clade A) exhibited a canonical structure, with three minor types, Ia, Ib, and Ic, identified due to modest shifts of border genes, *rps19*, *ndhF*, and *ycf1*, relative to the IR–SC boundaries. In comparison, type II (Clade B) revealed two inversions: a ∼21-kb inversion from *ndhB* to *ycf1* and a ∼6-kb inversion spanning *ycf1* alone. Furthermore, type III (Clade C) displayed a ∼14-kb inversion from *ndhB* to *trnR-ACG* compared with type I (Fig. 1b).

### Accumulated Repeats Associated with Hypervariable Structures

**Fig. 2.**
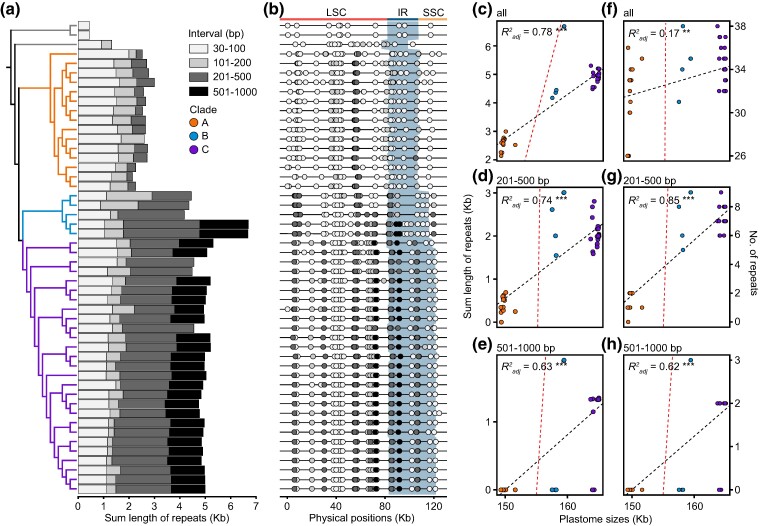
A repeat content within *Plantago* plastomes excluding one IR. a) Cumulative lengths of repeats categorized into four intervals. b) Distributions of repeats indicated by circles. LSC, IR, and SSC are represented with the bar on top. The blue shadows depict the IR. Regression analyses between plastome size and c) all, d) 201 to 500 bp, e) 501 to 1,000 bp cumulative repeat lengths. Correlations between plastome size and f) all, g) 201 to 500 bp and h) 501 to 1,000 bp cumulative repeat counts. The black and red dotted lines of best fit represent the linear model and the PGLS model, respectively. Statistical significance (**, *P* < 0.01; ***, *P* < 0.001) and coefficient of determination (*R*^2^_adj_) based on the standard linear model are presented.

*Plantago* exhibited a significantly higher number of repeats compared with the outgroups (Fig. 2a). Within *Plantago*, Clade B and Clade C accumulated a greater total length of repeats than Clade A (*P* = 2*e*−11 and 0.00013, respectively). However, this difference was not observed in terms of total repeat numbers (*P* = 0.058 and 0.017, respectively) or repeats shorter than 200 bp. Instead, it was predominantly due to the accumulation of repeats in the range of 201 to 500 bp (supplementary figs. S3 and S4 and supplementary table S2, Supplementary Material online). Notably, repeats exceeding 500 bp were exclusive to Clade B and Clade C (Fig. 2a). These longer repeats were distributed unevenly across the entire plastome (Fig. 2b). By comparing the similarities and locations, it was evident that the single longer repeats in Clade B and Clade C were not fusions of several short ones observed in Clade A. The cumulative lengths of repeats significantly contributed to variations in plastome size, particularly those exceeding 200 bp (Fig. 2c to e; supplementary fig. S4, Supplementary Material online). Although the linear standard model supported positive correlations between repeat counts and genome sizes, these correlations became weaker when using the PGLS method (Fig. 2f to h). The significantly strong phylogenetic signals revealed by the PGLS method indicated apparent clade-specific patterns in several genomic traits, including total size (*K* = 19.29, *P* = 0.001), IR size (*K* = 21.48, *P* = 0.001), SSC size (*K* = 21.90, *P* = 0.001), and GC content of IR (*K* = 23.09, *P* = 0.001) (supplementary table S3, Supplementary Material online). Additionally, certain repeats were positioned at IR inversion breakpoints or associated with genes exhibiting higher *d*_S_ (supplementary fig. S5, Supplementary Material online). Some repeats also produced the duplications of genes and exons as displayed in Fig. 1a.

**Fig. 3.**
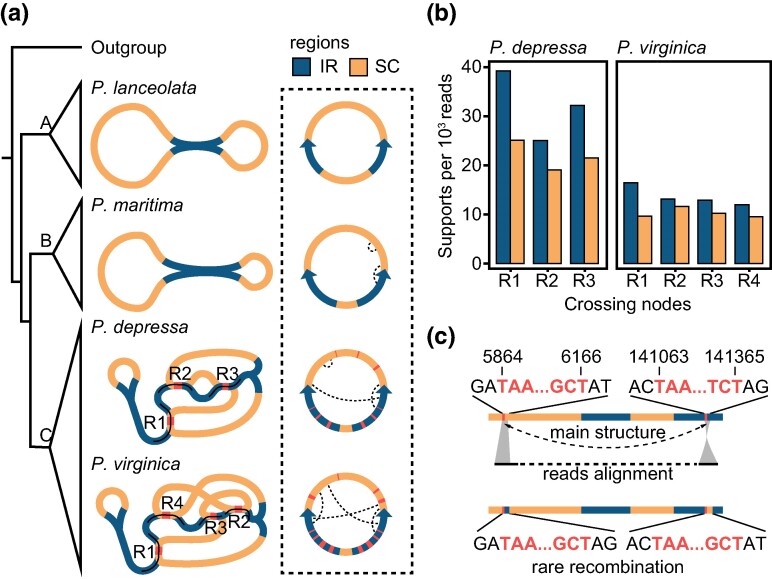
Structural complexity mediated by repeats. a) Schematic diagrams of plastomes during de novo assembly. The red regions denote the repeats (R1 to R4) present in Bandage as crossing nodes. Final forms of plastomes are displayed in the dotted frame, with black dotted curves joining locations of rare recombination caused by internal repeats within plastomes. b) Counts of plastome reads supporting connections in a. c) An example of rare recombination in *P. depressa* plastome. Repeats and flanking sequences are denoted in red bold and black font, respectively. Single reads aligned to breaking regions in the main structure are shown by black bars. The black curve indicates the recombination.

During the reference-free de novo assembly of plastomes, certain repeats in Clade C served as crossing nodes (R1 to R3 ranging from 137 to 579 bp in *Plantago depressa* and R1 to R4 ranging from 137 to 516 bp in *Plantago virginica*), leading to intricate networks that could encompass several potential alternative structures in the assembly graph of short reads (Fig. 3a). However, mapping of long reads did not confirm any recombination activity around these crossing nodes (Fig. 3b). This affirmed that these plastome networks resulted from the length limitations of short reads rather than genuine structural variants. Although short-read sequencing failed to identify substoichiometric (low-frequency) rearrangements, HiFi reads identified internal repeats (R1 to R4, depicted as the dotted curves) that facilitated two extremely infrequent recombination events, namely one within LSC and the other between LSC and IR, of *P. maritima* in Clade B, only with 0.13% (2/1,538 reads) and 0.15% (2/1,342 reads) recombination rates, respectively. Similarly, in Clade C, three and four pairs of repeats contributed to rare recombination in *P. depressa* (0.16% to 0.25%, 3/1,879 to 3/1,202 reads) and *P. virginica* (0.11% and 0.29%, 2/1,818 and 5/1,724 reads), respectively. For instance, in *P. depressa* from Clade C, a 303-bp repeat led to rare recombination between two regions located in LSC and IR, respectively (Fig. 3c). In contrast, no such recombination was observed in *Plantago lanceolata* in Clade A.

### Elevated Synonymous Substitution Rates in Clades with Large Inversions

**Fig. 4.**
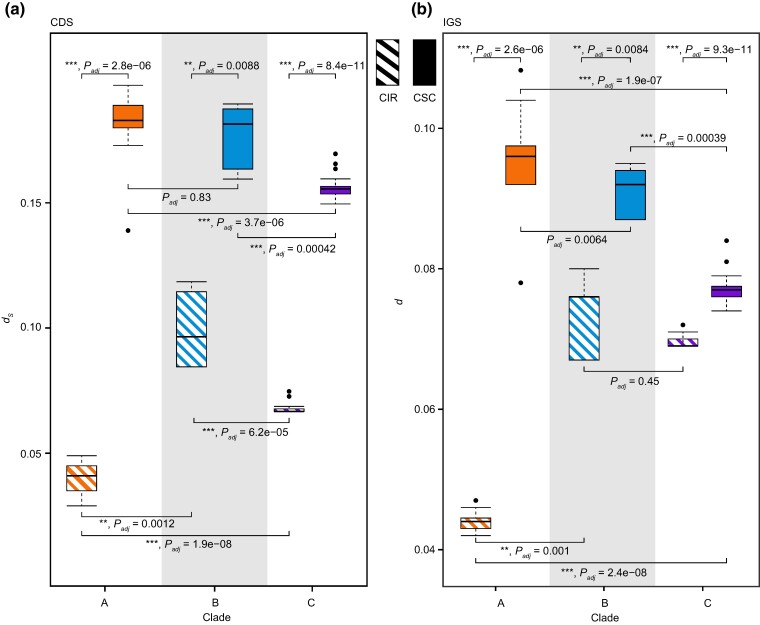
A *d*_S_ comparison between CSC and CIR. The boxes represent a) *d*_S_ for CDS concatenations and (b) *d* for IGS concatenations. Each box spans the first quartile to the third quartile (inter-quartile range [IQR]). The horizontal line across the box signifies the median value. The vertical line extends from the box to the highest value within 1.5 times the IQR. ** *P*_adj_ < 0.01; *** *P*_adj_ < 0.001 in Wilcoxon’s rank-sum test with a Bonferroni correction.

To assess variations in nucleotide substitution rates among three evolutionary clades, calculations were done for 66 shared PCGs and 76 intergenic spacers (IGSs) (longer than 150 bp excluding aligned gaps; supplementary table S5, Supplementary Material online), along with several concatenations. To mitigate regional effects, CDS and IGS were categorized as Consensus SC (CSC, consistently located in SC in all species), Consensus IR (CIR, consistently located in IR in all species), and Variable SC-or-IR (VSR, located in either SC or IR; Table 1; supplementary table S6, Supplementary Material online). Surprisingly, substitution rates of IGSs were often lower than those of CDSs but not always. For CIR, CDS exhibited higher rates than IGS in both Clade A and Clade B, but not in Clade C (*P* = 2.7*e*−05, 0.0084, and 0.8517, respectively; supplementary fig. S6a, Supplementary Material online). For CSC, CDS consistently displayed higher rates across all clades (*P* = 2.8*e*−06, 0.0088, and 2.2*e*−10, respectively; supplementary fig. S6b, Supplementary Material online). Furthermore, within each clade, CIR consistently displayed lower rates than CSC in both CDS (*P* = 2.8*e*−6, 0.0088, and 8.4*e*−11, respectively; Fig. 4a) and IGS (*P* = 2.6*e*−6, 0.0084, and 9.3*e*−11, respectively; Fig. 4b). Among the three clades, *d*_S_ for CSC was higher in Clade A and Clade B (A vs. B: *P* = 0.83) than in Clade C (A vs. C: *P* = 3.7*e*−6; B vs. C: *P* = 0.00042; Fig. 4). However, concerning *d*_S_ for IR, the pattern was Clade A < C < B (A vs. C: *P* = 1.9*e*−8; B vs. C: *P* = 6.2*e*−5; Fig. 4). Minor differences, such as Clade A having higher rates than Clade B for CSC (*P* = 0.0064) and Clade B and Clade C showing similar rates for CIR (*P* = 0.45), were observed in IGS. Thus, substantial changes were evident between CSC and CIR in Clade A.

**Table 1:** Genic locations in *Plantago* plastomes

Group	Gene
Consensus SC (CSC)	*accD, atpA, atpB, atpE, atpF, atpH, atpI, cemA, clpP, infA, matK, ndhC, ndhJ, ndhK, petA, petB, petD, petG, petL, petN, psaA, psaB, psbA, psbB, psbC, psbD, psbE, psbH, psbK, psbZ, rbcL, rpl14, rpl16, rpl20, rpl22, rpl33, rpoA, rpoB, rpoC1, rpoC2, rps11, rps12_e23, rps14, rps16, rps18, rps2, rps3, rps4, rps8, ycf3, ycf4, ccsA, ndhF* ^a^ *, rps19* ^a^ *, rpl32* ^a^
Consensus IR (CIR)	*ndhB, rpl2, rpl23, rps7, ycf2*
Variable SC-or-IR (VSR)	*ndhA, ndhD, ndhE, ndhG, ndhH, ndhI, psaC, rps15, ycf1*

Genes shorter than 150 bp are excluded.

^a^Genes across the IR–SC boundaries with fewer than 40 nucleotides in IR that are treated as CSC for convenience.

**Fig. 5.**
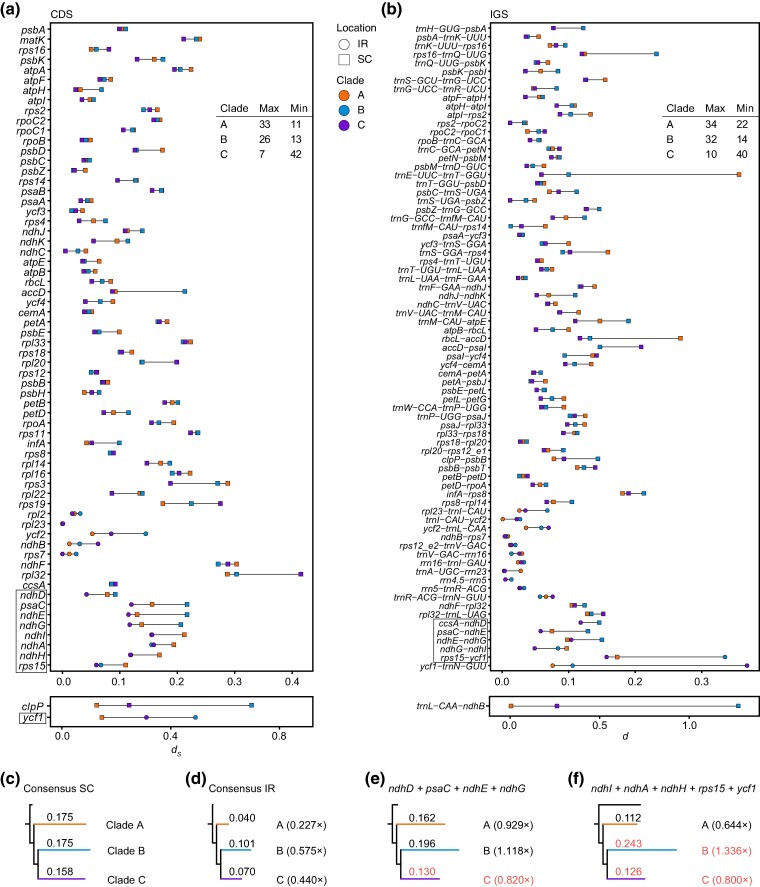
Comparisons of nucleotide substitution rate among loci of *Plantago* plastomes. The panels show changes in a) *d*_S_ among CDSs and b) *d* among IGSs. VSR loci are boxed. Loci situated in IR and SC are denoted by circles and squares, respectively. The estimation of *d*_S_ for concatenations of c) CSC and d) CIR is presented. The ratio of branch lengths is provided for the concatenations of e) *ndhD* + *psaC* + *ndhE* + *ndhG* and f) *ndhI* + *ndhA* + *ndhH* + *rps15* + *ycf1* relative to branch lengths in CSC. The branches containing VSR genes located in IR are highlighted in red.

Moreover, CSC was further categorized into seven functional groups to explore potential differences in *d*_S_ (supplementary table S7, Supplementary Material online). However, no statistically significant differences were detected between any pair (*P* > 0.05; supplementary fig. S7, Supplementary Material online), suggesting the decisive influence of the overall CSC gene set rather than specific functions. Among the 66 genes, 33 exhibited the highest *d*_S_ in Clade A, with 11 having the lowest. Conversely, only seven genes displayed the highest *d*_S_ in Clade C, while 42 had the lowest. Clade B was intermediate, with 26 having the highest and 13 the lowest genes (Fig. 5a; supplementary table S8, Supplementary Material online). Similar trends were observed for IGS nucleotide substitution rates, with 34 and 10 IGS having the highest rates in Clade A and Clade C, respectively. More IGSs had their lowest substitution rates in Clade C (40) than in the other two clades, a slight deviation from CDS patterns (Fig. 5b; supplementary table S9, Supplementary Material online), indicating a lower global rate in Clade C.

Certain genes, including *accD*, *clpP*, *rpl20*, and *ycf1*, exhibited higher *d*_S_ in Clade B and/or Clade C than in Clade A. These genes were situated near repeats or contained internal repeats. Additionally, *ndhB* and *ycf1* resided at the IR inversion breakpoints, both initially flanked inversion breakpoints (Clade A) with relatively low *d*_S_ but resided next to new gene neighbors after the inversion (Clade B and Clade C) with elevated *d*_S_, suggesting a strong association with inversion-mediated mechanisms possibly linked with repeats. Elevated rates in IGS at breakpoints were evident in *rps15-ycf1*, *ycf1-trnN-GUU*, *trnR-ACG-trnN-GUU*, and *trnL-CAA-ndhB*. Furthermore, *rps19* and *ndhF* at IR–SC junctions displayed relatively high *d*_S_ in all clades, potentially influenced by IR–border fluctuations (Fig. 5a and b; supplementary fig. S5, Supplementary Material online).

### Decreased Substitution Rates Following Translocation into IR

It was evident that eight out of nine VSR genes exhibited a decrease in *d*_S_, with the exception of *ycf1* (Fig. 5a). *d*_S_ were lower in CIR genes than in CSC genes in all three clades, but the difference was more pronounced in Clade A (0.227×) than in Clade B (0.575×) or Clade C (0.440×), indicating a more serious IR-dependent decrease in Clade A (Fig. 5c and d; supplementary table S10, Supplementary Material online). The *d*_S_ of VSR genes consistently decreased upon translocation into IRs. When translocating the genes into the IR of Clade C, the combination of *ndhD + psaC + ndhE + ndhG* declined to 0.820× of its CSC counterpart, while those retained in the SC regions of the other two clades maintained a similar level to their respective CSC (0.929× and 1.118×, respectively; Fig. 5e). These four individual VSR genes each showed a 0.265× to 0.757× deceleration in *d*_S_ in Clade C, slowing more substantially than in Clade A or Clade B, in which they remained in the SSC (supplementary fig. S8a to d, Supplementary Material online). However, *ndhI + ndhA + ndhH + rps15 + ycf1*, on the contrary, increased to 1.336× in Clade B after translocation, mainly due to the unexpected acceleration of *ycf1* (2.862×; Fig. 5f; supplementary fig. S8e to i, Supplementary Material online). Similarly, although *ndhI + ndhA + ndhH + rps15 + ycf1* was only 0.800× of CSC in Clade C, it was higher than the 0.644× in Clade A, again attributable to *ycf1* acceleration (1.807×; supplementary fig. S8i, Supplementary Material online).

### Increased GC Content After Translocation into IR

The higher GC content of ribosomal RNA (rRNA) genes contributed to an overall increase in GC content of IR in Clade A compared with Clade B and Clade C, due to a larger proportion of rRNA genes in relative smaller regions. To investigate alterations after translocation into the IR, the GC content of VSR CDS was systematically compared, revealing an increase in GC content across all cases. Similarly, translocated IGS exhibited a similar trend of increased GC content, except for two instances where a decrease was observed (*trnN-GUU-ndhF* and *psaC-ndhE*; supplementary fig. S9a, Supplementary Material online). Among CDSs, the third codon position consistently displayed lower GC content than the first and second positions and underwent more pronounced changes after translocation, suggesting a relaxation of GC-biased constraints compared with the first and second codon positions (supplementary fig. S9b to d, Supplementary Material online). relative synonymous codon usage (RSCU) for some translocated CDSs varied among genes and clades; for instance, *ndhA* in Clade C exhibited a preference for CAC to encode histidine, unlike the preference for CAU in Clade A and Clade B (supplementary fig. S10, Supplementary Material online). Additionally, in Clade C IR, 13 loci that always reside in IR across three clades experienced a GC content decrease, including two CDSs (*rps7* and *ycf2*), two tRNA genes (*trnN-GUU* and *trnR-ACG*), and nine IGSs (supplementary fig. S11, Supplementary Material online). Notably, 6 out of the 13 loci were situated around or in the vicinity of breakpoints.

## Discussion

### Accumulation of Larger Repeats Decreased Plastome Stability

In general, repeats in plastomes are fewer and shorter than those in land plant mitogenomes. Nevertheless, various studies have highlighted the connections between repetitive elements and the structural evolution of plant organellar genomes (Weng et al. 2013; Brázda et al. 2018; Cole et al. 2018; Wynn and Christensen 2019; Wang et al. 2024). Repeat-mediated homologous recombination (HR) is the primary mechanism, responsible for double-strand break (DSB) repair in plant organellar genomes, including both accurate (gene conversion) and less accurate (break-induced replication) processes based on the invasion of the lagging strand (Maréchal and Brisson 2010; Christensen 2018). Although limited proportions are provided in plastid genomes, empirical data indicated that nonhomologous events accounted for only 2.74% in a somatic hybrid mitochondrial recombination map (Gandini et al. 2023). To our knowledge, the plastid employs all types of HR repair mechanisms for DSB repair, in which the compromised related proteins lead to unexpected rearrangements and elevated point mutations, verified by additional experimental and bioinformatic analyses (Odahara et al. 2015a, 2015b; Wu et al. 2020b; Mahapatra et al. 2021; Zou et al. 2022). Within *Plantago* plastomes analyzed here, Clade B and Clade C exhibited both increased larger repeats, particularly those exceeding 200 bp, and significant inversions within the IR flanked by repeats (supplementary fig. S5, Supplementary Material online). This observation supports prior research suggesting a strong correlation between repeat locations and rearrangement hotspots (Chumley et al. 2006; Mower et al. 2021a) as well as breakpoints (Lee et al. 2023a; Xu et al. 2023), implying that these inversions were induced by repeats (Palmer and Thompson 1982). Although some repeats were not perfectly located at the endpoints of inversions, nor showed 100% identity within pairs, it is possible that some repeats might not be fully detected due to the chosen search criteria or may have evolved such that their size or sequence has not been maintained.

During the de novo reference-free assembly based on short reads, the aggregation of repeats, beyond the coverage of short reads, unexpectedly gave rise to a complex network referred to as “crossing nodes” (red regions in Fig. 3a). Typically, such complex configurations are encountered in plant mitogenomes characterized by extensive repeats, making structural resolution challenging. However, the validation based on HiFi reads confirmed the absence of actual recombination around these “crossing nodes” (Fig. 3a and b). Nonetheless, we observed exceptionally rare recombination events facilitated by repetitive elements (black dotted curves in Fig. 3a and c). Notably, these repeats that mediated rare recombination are not those hindering assembly in Fig. 3a. The relatively sporadic recombination mediated by small and intermediate repeats within plastomes constitutes a crucial mechanism in the dynamics of organellar genomes (Broz et al. 2022; Zou et al. 2022). Repeats with recombination activity contribute to structural rearrangements, giving rise to distinct genomic isomers with varying stoichiometric abundance within the intracellular milieu. These stoichiometric forms coexist and undergo dynamic shifts over time, known as SSS, as evidenced in certain species within family Cupressaceae (Guo et al. 2014; Qu et al. 2017; Lee et al. 2020), although the regulatory mechanisms governing this process in plastomes remain somewhat uncertain. SSS might be adaptive (Woloszynska 2009; Marina et al. 2023), contributing to genomic plasticity and potentially conferring adaptive advantages in response to changing environmental conditions. Whether these infrequent isomers in *Plantago* plastomes have consequential functional implications will require experimental analysis at the population level.

An additional outcome of the accumulation of repeats is the observed acceleration of nucleotide substitutions, a phenomenon documented in *Silene* and *Pelargonium* (Sloan et al. 2012b; Zhu et al. 2016). Notably, *Silene* species characterized as “fast” exhibited a higher abundance of repeats, in terms of both length and count, compared with their “slow” counterparts. Remarkably, no repeats exceeding 500 bp were identified in those *Silene* species, indicating potential distinctions in the processes influencing this acceleration when juxtaposed with the situation in *Plantago* (supplementary fig. S12, Supplementary Material online). Specifically, certain CDSs, including *accD*, *rpl20*, and *clpP*, were not strictly positioned at inversion breakpoints but in close proximity to repeats or even harbored repeats within their sequences (supplementary fig. S5, Supplementary Material online), suggesting some repeat-relevant influencers. Explanations for the acceleration observed in *accD* include a more permissive purifying selection on repeats and neighboring regions or a heightened frequency of nucleotide substitutions in the flanking regions induced by repeats autonomously (Park et al. 2017; Li et al. 2018). However, the extent to which these explanations might apply to other instances of acceleration remains unclear, leaving the possibility of additional (non)adaptive evolutionary mechanisms at play. For instance, in certain scenarios, the acceleration observed in *accD* and *clpP* coincides with other components within a large complex that is subjected to selection pressures, implying co-evolution to maintain the assembled complex, i.e. acetyl-CoA carboxylase and ATP-dependent Clp protease (Erixon and Oxelman 2008; Williams et al. 2019; Abdel Ghany et al. 2022). Retroprocessing has been hypothesized to elevate substitutions in many lineages including *Plantago* (Zhu et al. 2016). Here, however, no convincing variations in predicted RNA editing sites were observed in these intron-loss genes (*clpP*, *rpl2*, and *rpl16*) among the three clades (supplementary table S4, Supplementary Material online), rejecting an obvious role for intrageneric clade-specific mutagenic retroprocessing. Such retroprocessing tends to cause simultaneous loss of introns and retention of edited sites in organellar genome upon posttranscriptional processing, as observed in *atpF* in *Manihot esculenta* (Daniell et al. 2008) and more so in plant mitogenomes such as the genes *cox2*, *nad1*, and *nad4* in *Isoetes engelmannii* (Grewe et al. 2010), *nad7* and *cox2* in some *Silene* species (Sloan et al. 2010), and the entire mitogenome of *Welwitschia*, which experienced a massive loss of both edited sites and *cis*-spliced introns (Guo et al. 2016).

Correlations between increased substitution rates, cumulative repeats, and active rearrangements are evident in numerous plant lineages (Guisinger et al. 2008; Weng et al. 2013), and various hypotheses have been proposed to explain these correlated variations in plant organellar genomes. In *Oenothera* plastomes, an increase in spontaneous mutations is attributed to DNA replication slippage causing indels resulting from changes in copy numbers of tandem repeats (Massouh et al. 2016). While imperfect tandem repeats with indels are identified in a few prominent genes such as *accD* and *ycf1* in *Plantago*, the widespread distribution of nucleotide polymorphisms concurrently challenges the notion of replication slippage as the predominant, or at least not the sole, explanation. An intriguing explanation for the observed phenomena points toward potential dysfunction in nuclear-encoded genes associated with DNA replication, recombination, and repair (RRR) processes (Maréchal and Brisson 2010; Sloan et al. 2012a; Chevigny et al. 2020). This hypothesis gains support from the significant mitogenomic accelerations noted in species belonging to Clade B and Clade C (Cho et al. 2004; Mower et al. 2007, 2021b), although the complete *Plantago* mitogenomes are unavailable yet. In the case of *Plantago*, this acceleration might be attributed to one or more clade-specific dysfunctions in dual-targeted RRR genes. Notable candidates include, but are not limited to, MUTS homologue 1 (*MSH1*), RecA homologues 2 (*RecA2*), organellar single-stranded DNA-binding protein 3 (*OSB3*), Whirlye (*Why*), and *RadA* (Odahara et al. 2015a; Chevigny et al. 2020; Wu et al. 2020b; Gandini et al. 2023; Lee et al. 2023b). Alternatively, more complex scenarios, such as simultaneous loss of some plastid- and mitochondrion-targeted RRR genes, or disruptions in posttranscriptional processes hindering their normal function, could be at play (Gualberto and Newton 2017; Xiang et al. 2022). In many instances, RRR genes act as surveillance mechanisms, suppressing recombination and ensuring accurate DNA damage repair. This has been corroborated by the absence of RRR genes in highly rearranged lineages and mutant strains exhibiting an increase in point mutations and structural rearrangements (Odahara et al. 2015b; Wu et al. 2020b; Zou et al. 2022; Lee et al. 2023b). Investigating nuclear genomes of *Plantago* species will contribute to our standing of such potential regulations.

### Multiple Factors Shaped Substitution Rate Changes After IR Expansion

IRs, which provide twice the template copies of SC, enhance efficiency in correcting internal mutations through a copy-dependent error correction mechanism, known as biased gene conversion (Wu and Chaw 2015). Theoretically, this process helps maintain lower nucleotide substitution rates and higher GC content in the IR, a pattern broadly observed across plant lineages (reviewed by Mower and Vickrey (2018)). Intriguingly, typical IRs can transform into uncanonical direct repeats via large-scale inversions in some *Selaginella* plastomes, and these direct repeats still function in copy-dependent repair to suppress substitution rates within repeats (Mower et al. 2019). This study made the same observations that CIR genes and IGS showed much lower substitution rates than their CSC counterparts, and GC content increased in most VSR loci (Fig. 4; supplementary fig. S9, Supplementary Material online). An interesting implication of the copy-dependent repair mechanism is that when loci translocate from SC regions into IRs, they should undergo rate deceleration, and vice versa. Confirming this expectation, all VSR loci in Clade B and Clade C of *Plantago*, with a few exceptions such as *ycf1* and *rps15-ycf1*, consistently decelerated upon translocating into IR, albeit to varying degrees (Fig. 5c; supplementary tables S8 and S9, Supplementary Material online). This IR-dependent deceleration has been widely observed in studies of angiosperms, gymnosperms, and ferns (Li et al. 2016; Zhu et al. 2016). Although some exceptions do exist, such as the IR-to-SC *ycf2* in *Ginkgo biloba* still retained a relatively lower rate, this was postulated to occur recently, making it unlikely to accumulate enough mutations (Lin et al. 2012).

Nonetheless, the accelerated evolution of certain loci within IR, such as *ycf1* and *ndhB*, along with several IGSs, cannot be solely explained by restricted evolutionary time, although *ycf1* showed high substitution rates across many lineages, indicating at least in part a locus-specific acceleration (Dong et al. 2015). Alternatively, a tempting explanation is the application of the localized hypermutation hypothesis, involving DSB and error-prone repair (Magee et al. 2010; Mower et al. 2021a). The genomic location of *ycf1* and *ndhB* near IR inversion breakpoints supports this hypothesis. In particular, *ycf1* was involved twice in the two independent inversions in Clade B, thereby accelerating more than in Clade C, which inversed only once (Fig. 5a). Accelerations caused by localized hypermutation were evident in relevant IGS as well such as *trnL-CAA-ndhB*, *trnR-ACG-trnN-GUU*, and *rps15-ycf1* (Fig. 5b). This phenomenon suggests that the IR-dependent decelerating effect can be suppressed or offset by the accelerating power of localized hypermutation. Furthermore, CDS in Clade B and Clade C exhibited a tendency to decline less than in Clade A when comparing CIR with CSC genes, implying a potentially looser IR-dependent restriction in the two clades with significant repeat accumulations (Fig. 5c and d).

## Conclusion

This study presented a comprehensive analysis of the plastome evolution in *Plantago*, revealing clade-specific variations in composition and organization. The observed alterations in plastome size, repeat content, and structural features provided valuable insights into the dynamic evolutionary processes shaping these plastomes. Notably, the expansion of and inversion in the IR, coupled with variations in large repeat accumulation, emerged as prominent contributors to plastome diversity. The intricate relationship between repeats, structural rearrangements, and nucleotide substitutions underscored the complex interplay of evolutionary forces. The roles of repeats in augmenting structural complexity and increasing evolutionary rates in *Plantago* plastomes were elucidated. The translocated loci were influenced by a combination of locus-specific effects, IR-dependent deceleration, and localized hypermutation acceleration. Nuclear-encoded RRR genes were posited as crucial underlying mechanisms for the overall observed variations. While this study advanced our understanding of repeat-mediated effects on plastome evolution in *Plantago*, some questions remain unanswered. Future research should explore the specific mechanisms driving repeat-induced structural rearrangements and nucleotide substitutions. The potential role of RRR genes as key regulators demands further investigation, including the exploration of their presence and functionality in *Plantago* nuclear genomes. Additionally, the study sets the stage for examining the population-level dynamics of plastome variations, considering the potential adaptive significance of the observed complexities. Overall, the evolving landscape of *Plantago* organellar genome research holds promise for uncovering additional nuances and contributing to a more comprehensive understanding of plant organellar genome evolution in fast-evolving clades.

## Materials and Methods

### Plant Materials and Genome Sequencing

We obtained a total of 75 plastomes representing 45 species and 2 subspecies of *Plantago asiatica* that cover all four subgenera and 16 sections within the *Plantago* genus (Plantaginaceae). Among them, 30 plastomes were sourced from GenBank (https://www.ncbi.nlm.nih.gov/nuccore/), and 14 were reassembled based on Illumina reads from Hassemer et al. (2019). We sequenced additional 31 plastomes from 19 species, with nine species being sequenced for the first time (supplementary fig. S1 and supplementary table S1, Supplementary Material online). For the newly sequenced plastomes, total genomic DNA was extracted from fresh or silica-dried leaves by a cetyltrimethylammonium bromide-based protocol (Doyle and Doyle 1987). The DNA was then sheared to fragments of approximately 300 bp by sonication with a Covaris E220 (Covaris, Brighton, UK). Subsequently, a DNA library was prepared and sequenced to generate 150-bp paired-end (PE) reads with the DNBSEQ platform at BGI (Shenzhen, China). Long-read sequencing was performed for four representative species, *P. lanceolata*, *P. depressa*, *P. virginica* and *P. maritima*, with the Pacific Biosciences (PacBio) High-Fidelity (HiFi) sequencing platform on Sequel II Systems (Pacific Biosciences of California, Inc., USA). Continuous long reads of *P. ovata* were acquired from Herliana et al. (2023) and corrected with NextDenovo v2.5.2 (https://github.com/Nextomics/NextDenovo).

### Plastome Assembly and Annotation

PE reads underwent quality assessment by FastQC v0.11.9 (https://kb.ndsu.edu/fastqc) and were trimmed in Trimmomatic v0.39 (Bolger et al. 2014). Given the high copy number of the plastome, reads were randomly extracted with SeqKit v2.1.0 (Shen et al. 2016) and de novo assembled in SPAdes v3.13.0 (see supplementary table S1, Supplementary Material online for the total number of bases used in each assembly). The assembly graph was simplified in Bandage v0.8.1 (Wick et al. 2015). To ensure integrity, PE reads were remapped against the plastome assembly with BWA-MEM v0.7.17 (https://github.com/lh3/bwa), and for the four representative species, further verification was conducted by remapping long reads with minimap2 v2.24 (Li 2018). Annotation of PCGs, tRNA genes, and rRNA genes was carried out in GeSeq (https://chlorobox.mpimp-golm.mpg.de/geseq.html) (Tillich et al. 2017), with references covering all subgenera, followed by necessary manual corrections. Schematic diagrams were generated in OGDRAW v1.3.1 (Greiner et al. 2019). The sequences were submitted to GenBank with accession numbers OR911464 to OR911494 (supplementary table S1, Supplementary Material online).

### Size, GC Content, Codon Usage, Repeat Analyses, and RNA Editing Prediction

Size and GC content of the total and each unit including LSC, SSC, and IRs were summarized through a custom Perl script (https://github.com/hwc2021/). Repeat identification was conducted with BLASTn v2.11.0+ (Ye et al. 2006) with a word size of 7 and an *e*-value cutoff of 1*e*−6, excluding one IR. Overlapping repeats were merged with BEDtools v2.30.0 (Quinlan and Hall 2010). Statistical differences were assessed by the Wilcoxon rank-sum test (Perolat et al. 2015) in which *P* values were adjusted with a Bonferroni correction. Regressions between repeats of different sizes and GC content were analyzed by Pearson’s correlation coefficient and PGLS in R v4.2 (Wilkinson 2011; Brokaw and Smotherman 2020). In PGLS analyses, Blomberg’ *K* < 1 indicates that closely related species are less similar to each other than expected by Brownian motion, while *K* > 1 means that closely related species are more similar to each other than expected. Recombination mediated by repeats longer than 30 bp was detected based on HiFi reads via Bandage v0.8.1 and HiFi-SR (https://github.com/zouyinstein/hifisr) (Zou et al. 2022), in which only those reads with full-length mapping to the plastomes were considered to avoid possible interference from nuclear plastid transfers. The RSCU was summarized in DAMBE v5.2 (Xia et al. 2003) and visualized in Hiplot (https://hiplot.org) (Li et al. 2022). RNA reads mapping was utilized for *P. lanceolata* in Clade A. For *Plantago coronopus* and *Plantago macrorhiza* in Clade B and *P. virginica* and *P. depressa* in Clade C, RNA editing sites were computationally predicted with CURE-chloroplast (Du et al. 2009) and PREPACT v2.0 (Lenz and Knoop 2013). In PREPACT, BLASTX prediction was utilized with *Nicotiana tabacum* (NC_001879.2) plastid as the reference, and the results were filtered with a 0.001 *e*-value cutoff and a 30% threshold for hits. All parameters in CURE-chloroplast were set to default.

### Collinearity Comparison

Rearrangements among the 47 plastomes were detected with the progressiveMauve algorithm (Darling et al. 2010), with a seed weight and the minimum locally collinear block calculated automatically. Collinearity in four representative example species was compared with NgenomeSyn (He et al. 2023).

### Phylogenetic Reconstruction

MAFFT v7.49 (Katoh et al. 2017) was employed to align the 75 whole plastomes with default settings, followed by trimming in Gblocks v0.91 (Talavera and Castresana 2007) to eliminate poorly aligned regions. Nucleotide substitution saturation for trimmed alignments was tested with DAMBE v5.2. *Aragoa abietina* (MW877561.1), *Aragoa cleefii* (MW877562.1), and *Littorella uniflora* (MW877563.1) were selected as outgroups for phylogeny reconstruction. A maximum likelihood (ML) tree was constructed with IQ-TREE v2.2.0-beta (Nguyen et al. 2015) under the UNREST + FO + R4 nucleotide substitution model, determined by the inbuilt ModelFinder (Kalyaanamoorthy et al. 2017), with 1,000 nonparametric bootstrap replicates to evaluate clade credibility. Forty-seven plastomes including one for each species/subspecies were further analyzed (supplementary fig. S1 and supplementary table S1, Supplementary Material online). Common CDSs were extracted and aligned for concatenation in MAFFT v7.49 with the codon pattern. The incongruence length difference test was conducted in PAUP v4.0 b10 (Swofford 2002), where *P* > 0.05 allowed CDS concatenation. An ML tree for 47 *Plantago* species was based on the GTR + I + G4 + F nucleotide substitution model with 1,000 nonparametric bootstrap replicates and visualized in iTOL v6.4 (Letunic and Bork 2021).

### Substitution Rate Estimation

To explore changes in the relative synonymous substitution rate within a phylogenetic framework, a model-based approach (model = 1) was employed with codeML in PAML v4.8 (Yang 2007) for both individual CDS and concatenated sequences. The ML tree, based on the concatenation of CDS, served as a constrained phylogeny. Gaps in alignment matrices were excluded (clean data = 1). The F3 × 4 model determined codon frequencies, and the GY94 codon substitution model was applied to estimate transition/transversion and *d*_N_/*d*_S_. Considering the absence of all *ndh* genes except *ndhE* in *L. uniflora*, a subtree from the CDS-based ML tree, excluding *L. uniflora*, was utilized to estimate the *d*_S_ for these *ndh* genes. Similarly, each common IGS was aligned and then trimmed in Gblocks v0.91 to obtain conserved sequences. IGSs at inversion breakpoints in the IR were identified and named based on the IGS in Clade A. Because it was difficult to accurately define breakpoints for those IGSs that contained different regional sequences due to inversions, the IGSs were aligned to find the appropriate breakpoints (based on typical sequences), and continuous well-aligned regions were kept for calculation. It is important to note that *trnN-GUU-ndhF* was excluded due to its absence in the other two clades, resulting from *ycf1* pseudogenization at the junction of IR_B_-SSC in Clade A. Cumulative branch lengths from the root to each terminal tip were summarized with Castor v1.7.11 in R v4.2. The REV nucleotide substitution model (model = 7) was employed for nucleotide substitution rate (*d*) estimation. Gaps in alignment matrices were excluded (clean data = 1). Differences among rates were assessed by using the Wilcoxon rank-sum test.

## Supplementary Material

evae177_Supplementary_Data

## Data Availability

The NCBI accession numbers of all used plastomes are provided in supplementary table S1, Supplementary Material online.
