# Supplementary material for: Accumulation of Large Lineage-Specific Repeats Coincides with Sequence Acceleration and Structural Rearrangement in Plantago Plastomes
Source: Genome Biol Evol. 2024 Aug 27;16(8):evae177. doi: 10.1093/gbe/evae177 (PMC11354287; doi:10.1093/gbe/evae177)
Supplement: evae177_Supplementary_Data [file evae177_supplementary_data.zip › Suppl. Figures.R2.pdf]

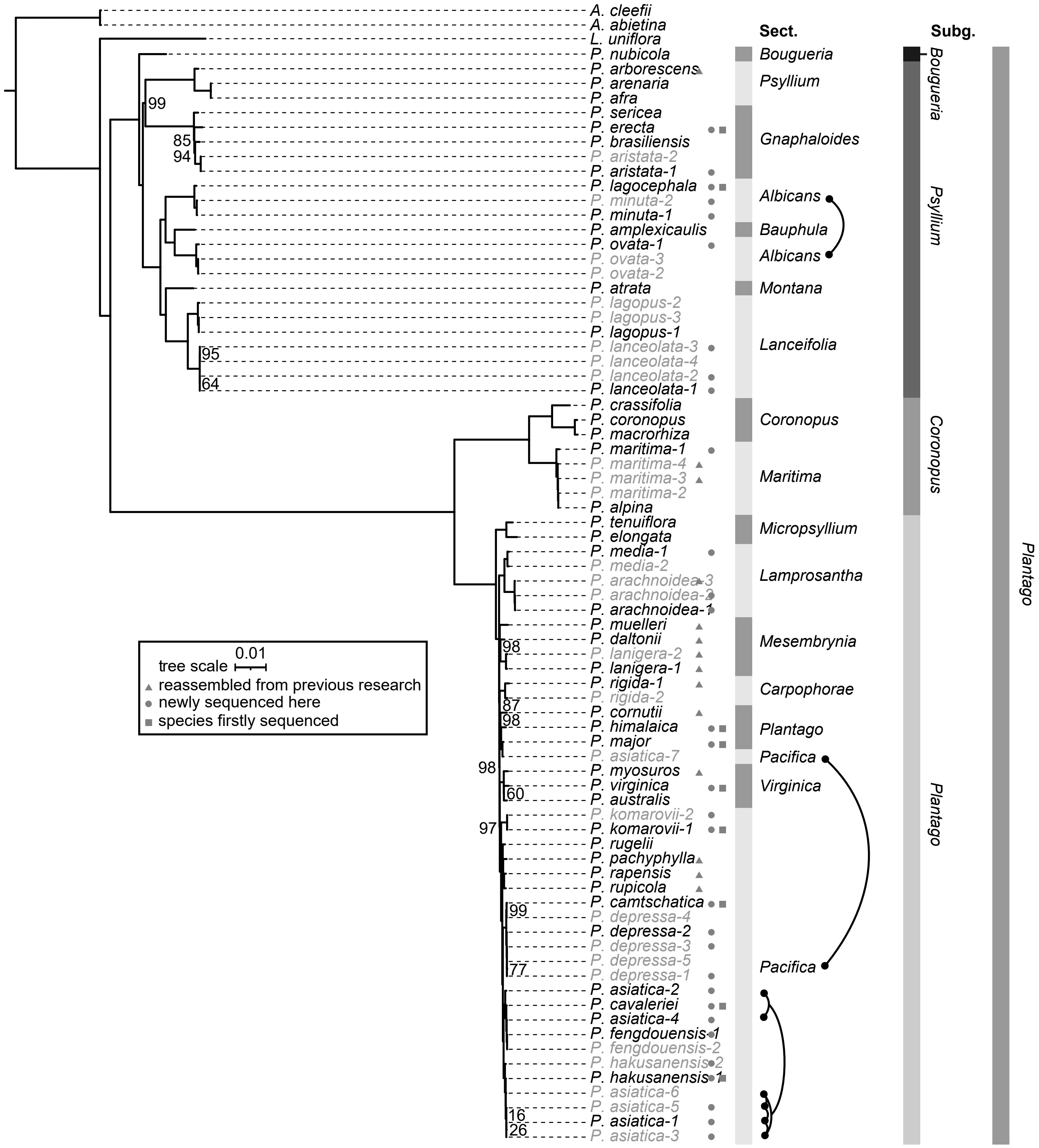

Fig. S1 A maximum likelihood tree based on 75 *Plantago* plastomes. Branch support is shown by the numbers above nodes from 1000 bootstrap replicates. Taxa excluded from the data set in subsequent phylogenetic analyses are shown in gray. Curves show the separated samples of the same species.

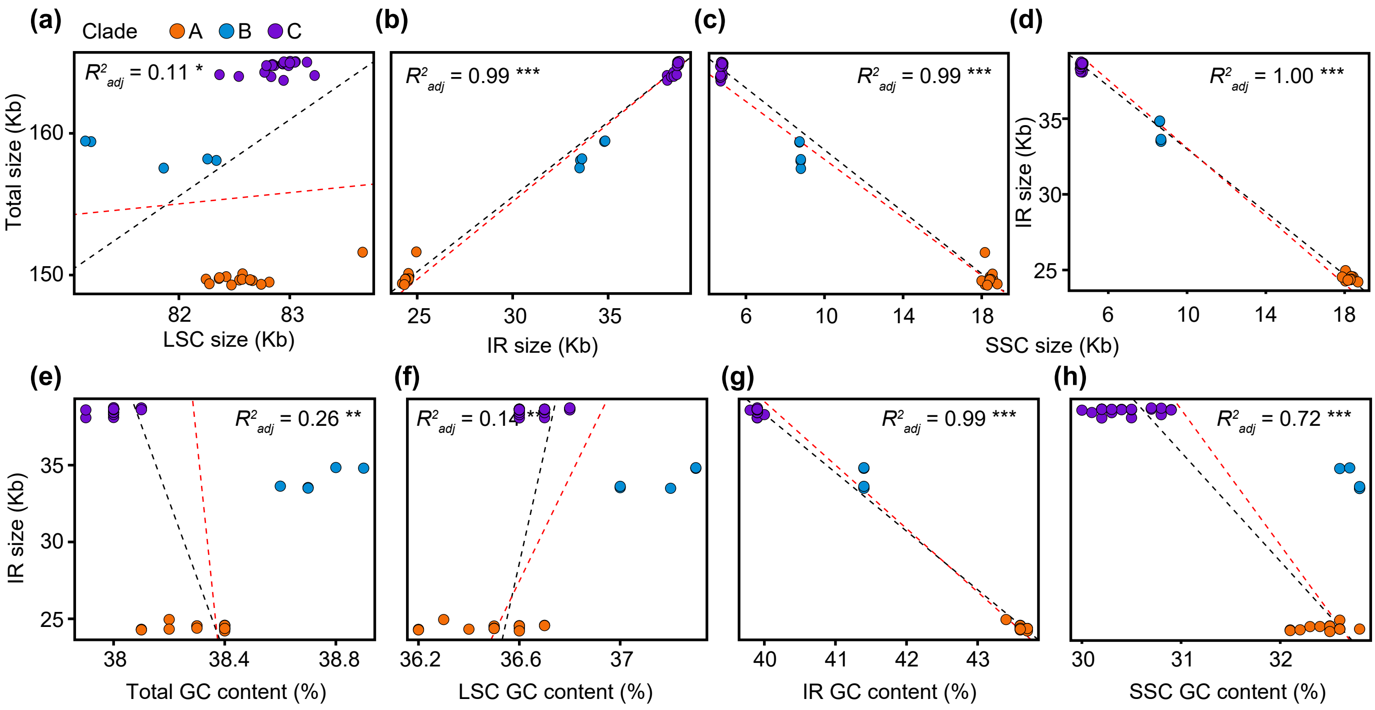

Fig. S2 Regressions between size and GC content of total and units. (a–c) Regressions between sizes in total and **(a)** large single copy (LSC), **(b)** inverted repeat (IR), and **(c)** small single copy (SSC). **(d)** Regressions between IR sizes of IR and SSC. (e–h) Regressions between IR sizes and GC content in **(e)** total, **(f)** LSC, **(g)** IR, and **(h)** SSC. Each circle represents a species coloured consistently with Fig. 2a. Black dotted lines of best fit represent the linear model and red dotted lines represent the PGLS model. Significance (*, $P < 0.05$; **, $P < 0.01$; ***, $P < 0.001$) and coefficient of determination (R^2_{adj}) based on the Pearson method are shown.

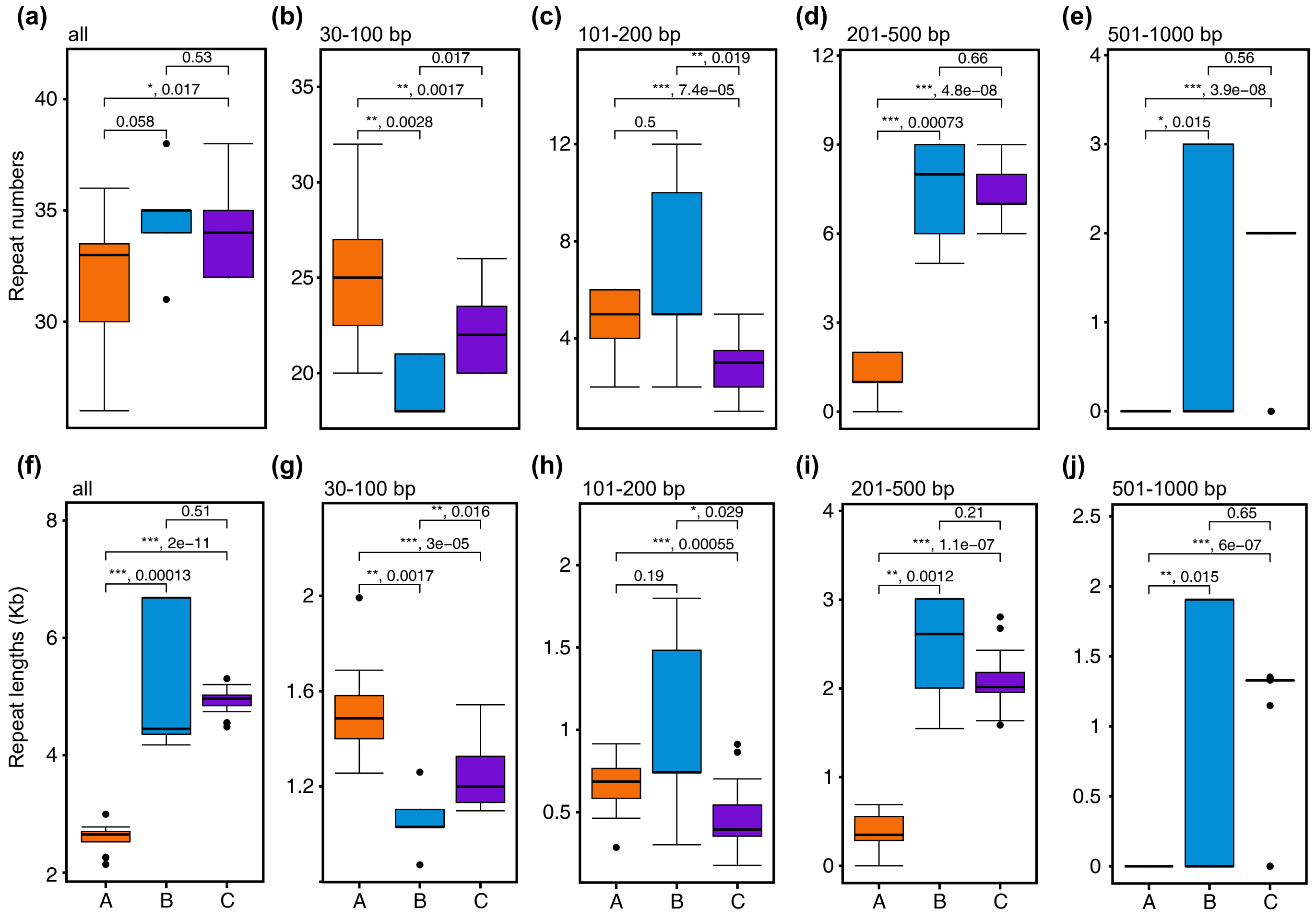

Fig. S3 Comparisons of the repeats among three evolutionary clades. Differences in numbers of **(a)** all, **(b)** 30–100 bp, **(c)** 101–200 bp, **(d)** 201–500 bp, and **(e)** 501–1000 bp repeats. Differences in accumulative lengths of **(f)** all, **(g)** 30–100 bp, **(h)** 101–200 bp, **(i)** 201–500 bp, and **(j)** 501–1000 bp repeats. The box spans the first quartile to the third quartile (IQR, inter-quartile-range). The horizontal line through the box denotes the median. The vertical line extends from the box to the highest value that is within 1.5 IQR. *, $P_{adj} < 0.05$; **, $P_{adj} < 0.01$; ***, $P_{adj} < 0.001$ in Wilcoxon rank sum test with a Bonferroni correction.

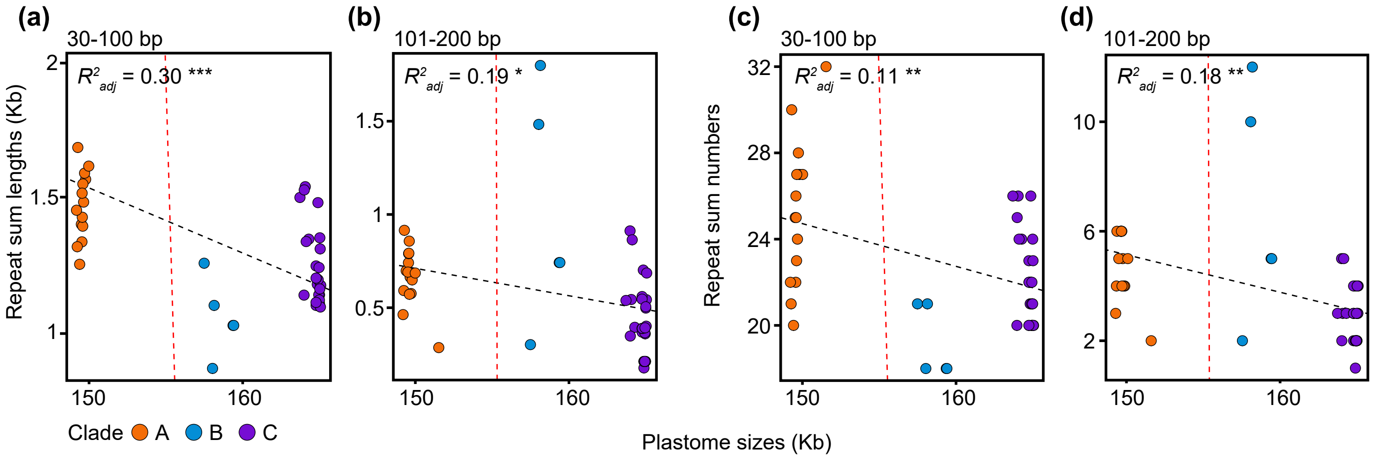

Fig. S4 Regressions between repeats and total size. Regressions between plastome size and **(a)** 30–100 bp and **(b)** 101–200 bp accumulative repeat lengths. Correlations between plastome size and **(c)** 30–100 bp and **(d)** 101–200 bp accumulative repeat counts. Each circle represents a species coloured consistently with Fig. 2a. Black dotted lines of best fit represent the linear model and red dotted lines represent the PGLS model. Significance (**, $P < 0.01$) and coefficient of determination (R^2_{adj}) based on the Pearson method are shown.

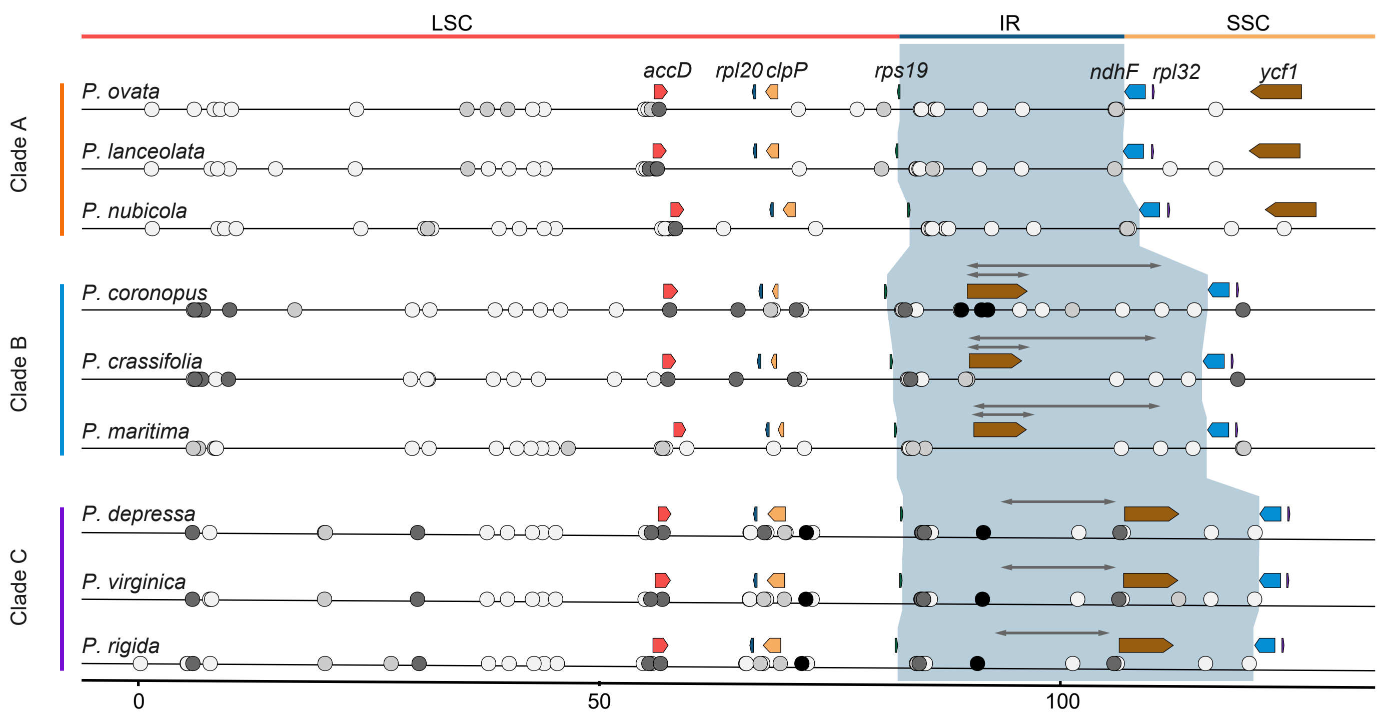

Fig. S5 Examples of inversion breakpoints and genes with high d_S flanked by repeats. Circles denote repeat classified into four length intervals (30–100 bp, light gray; 101–200 bp, gray; 201–500 bp, dark gray; 501–1000 bp, black). LSC (red), IR (blue), and SSC (orange) are shown with the bar on top. Blue shadows show the IR. Lines with arrows indicate inversions.

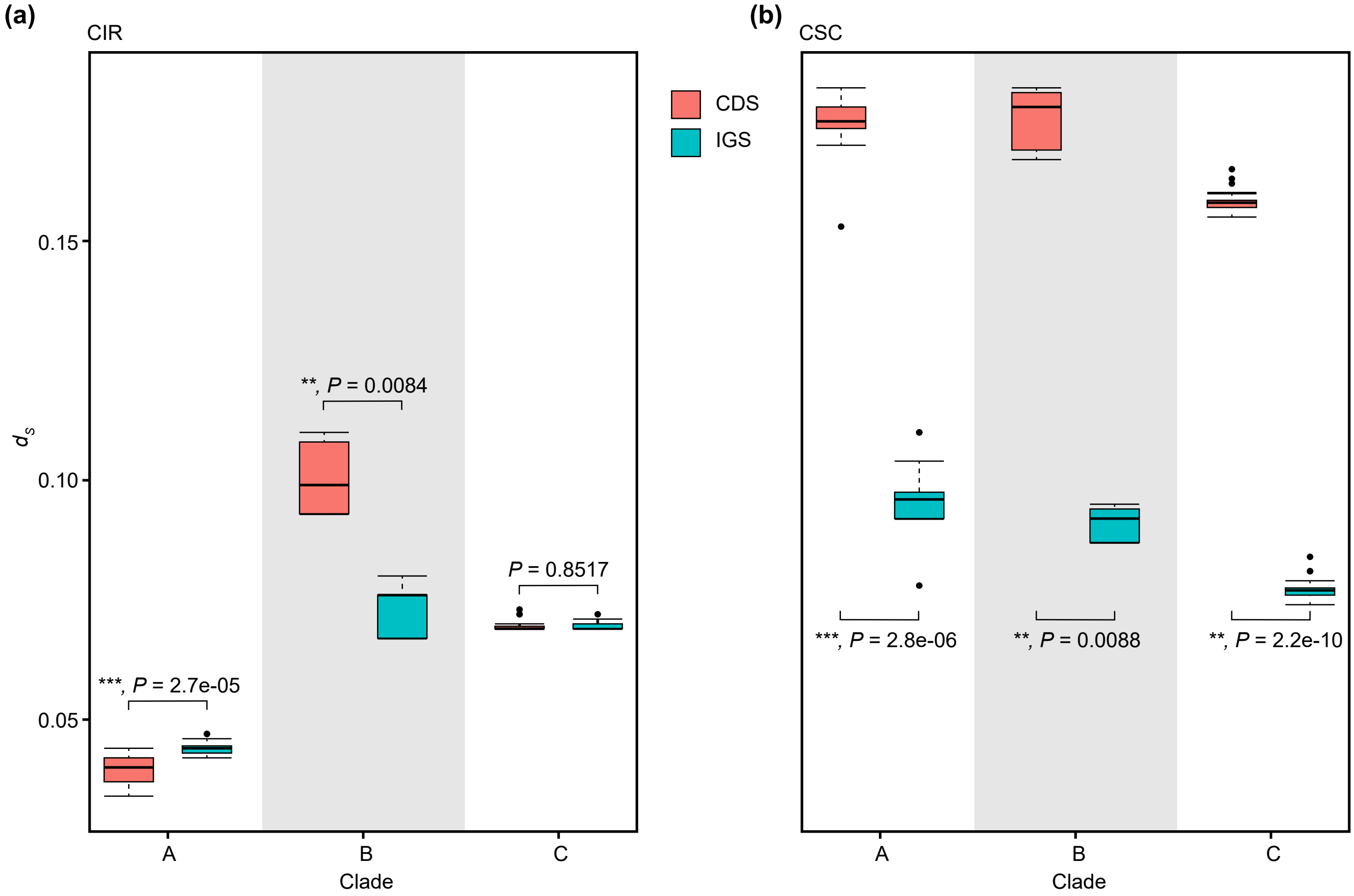

Fig. S6 Comparison of rates for CDS and IGS of (a) CIR and **(b)** CSC. The box spans the first quartile to the third quartile (IQR, inter-quartile-range). The horizontal line through the box denotes the median. The vertical line extends from the box to the highest value that is within 1.5 IQR. **, $P_{adj} < 0.01$; ***, $P_{adj} < 0.001$ in Wilcoxon rank sum test with a Bonferroni correction.

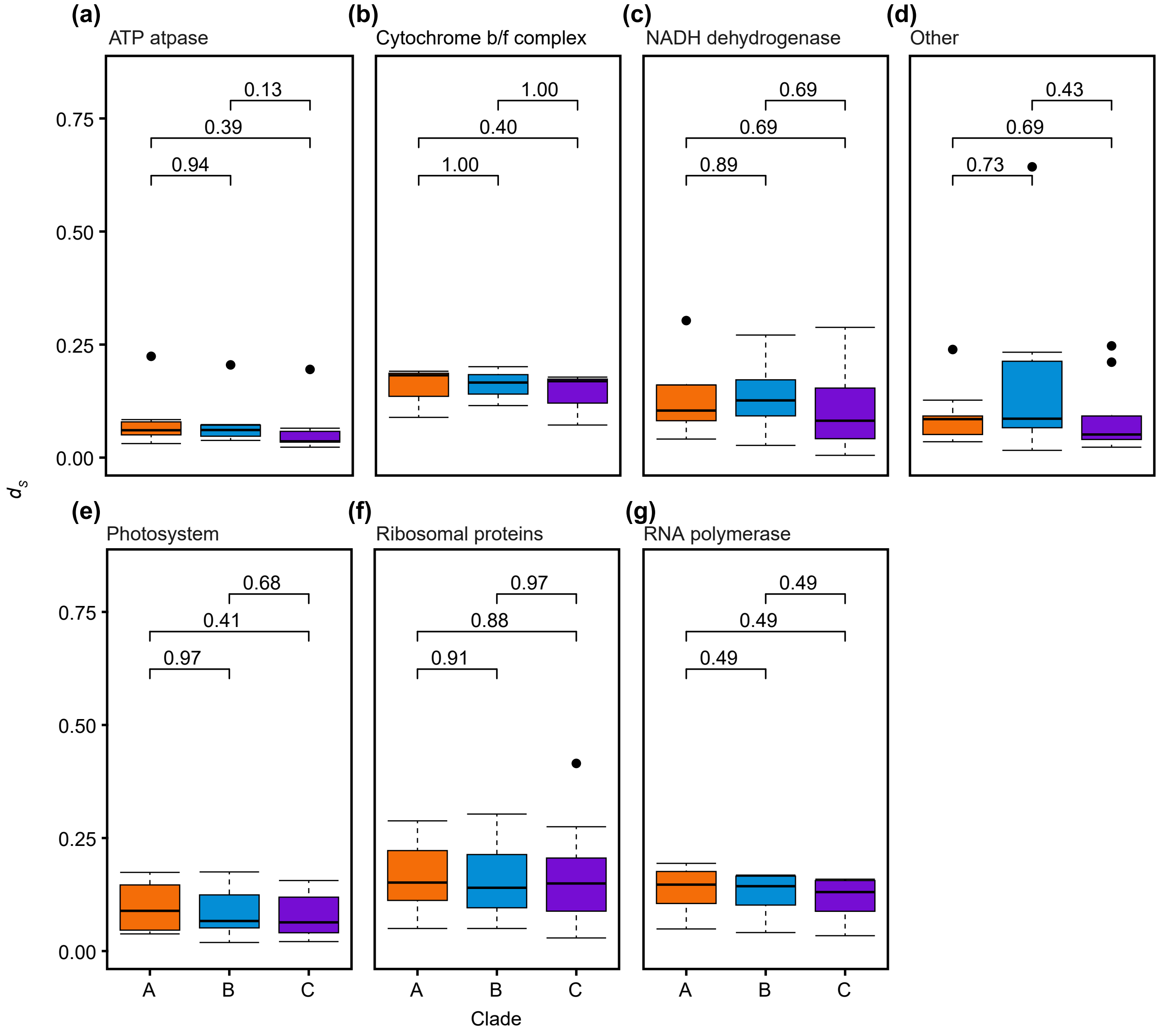

Fig. S7 Comparison of d_S for CSC genes among functional groups. (a) ATPase; **(b)** Cytochrome b/f complex; **(c)** NADH dehydrogenase; **(d)** Other; **(e)** Photosystem; **(f)** Ribosomal proteins; and **(g)** RNA polymerase. The box spans the first quartile to the third quartile (IQR, inter-quartile-range). The horizontal line through the box denotes the median. The vertical line extends from the box to the highest value that is within 1.5 IQR. **, $P_{adj} < 0.01$; ***, $P_{adj} < 0.001$ in Wilcoxon rank sum test with a Bonferroni correction.

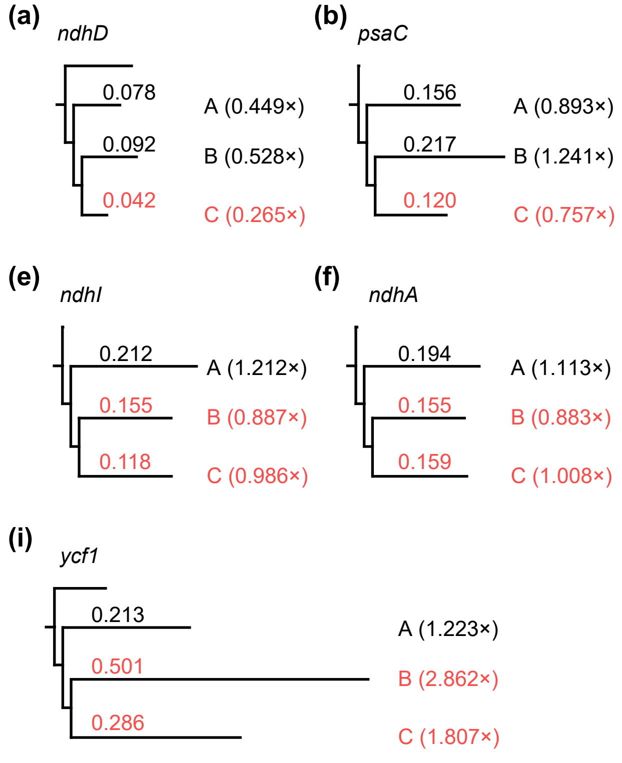

Fig. S8 Changes of d_S for individual VSR relative to CSC genes. A ratio of branch lengths ($d_{S)}$ is given for **(a)** *ndhD*, **(b)** *psaC*, **(c)** *ndhE*, **(d)** *ndhG*, **(e)** *ndhI*, **(f)** *ndhA*, **(g)** *ndhH*, **(h)** *rps15*, and **(i)** *ycf1* relative to branch lengths in Consensus SC. Branches with Variable SC-or-IR genes located in IR are in red.

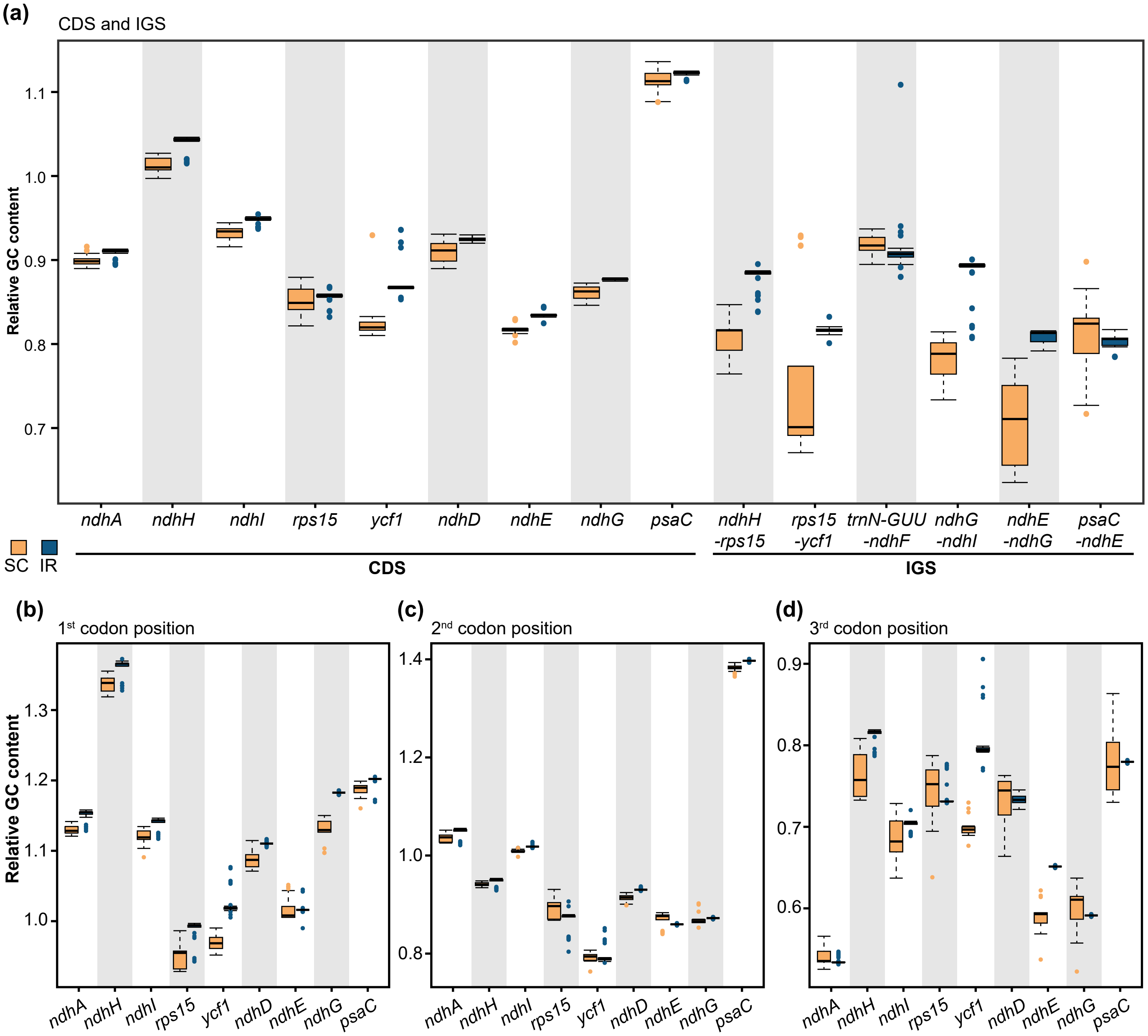

Fig. S9 Changes of relative GC content (divided by total GC content) among relocated sequences and codon positions. (a) Relative GC content of relocated CDS and IGS. Relative GC content of **(b)** the first, **(c)** second, and **(d)** third codon positions of relocated CDS.

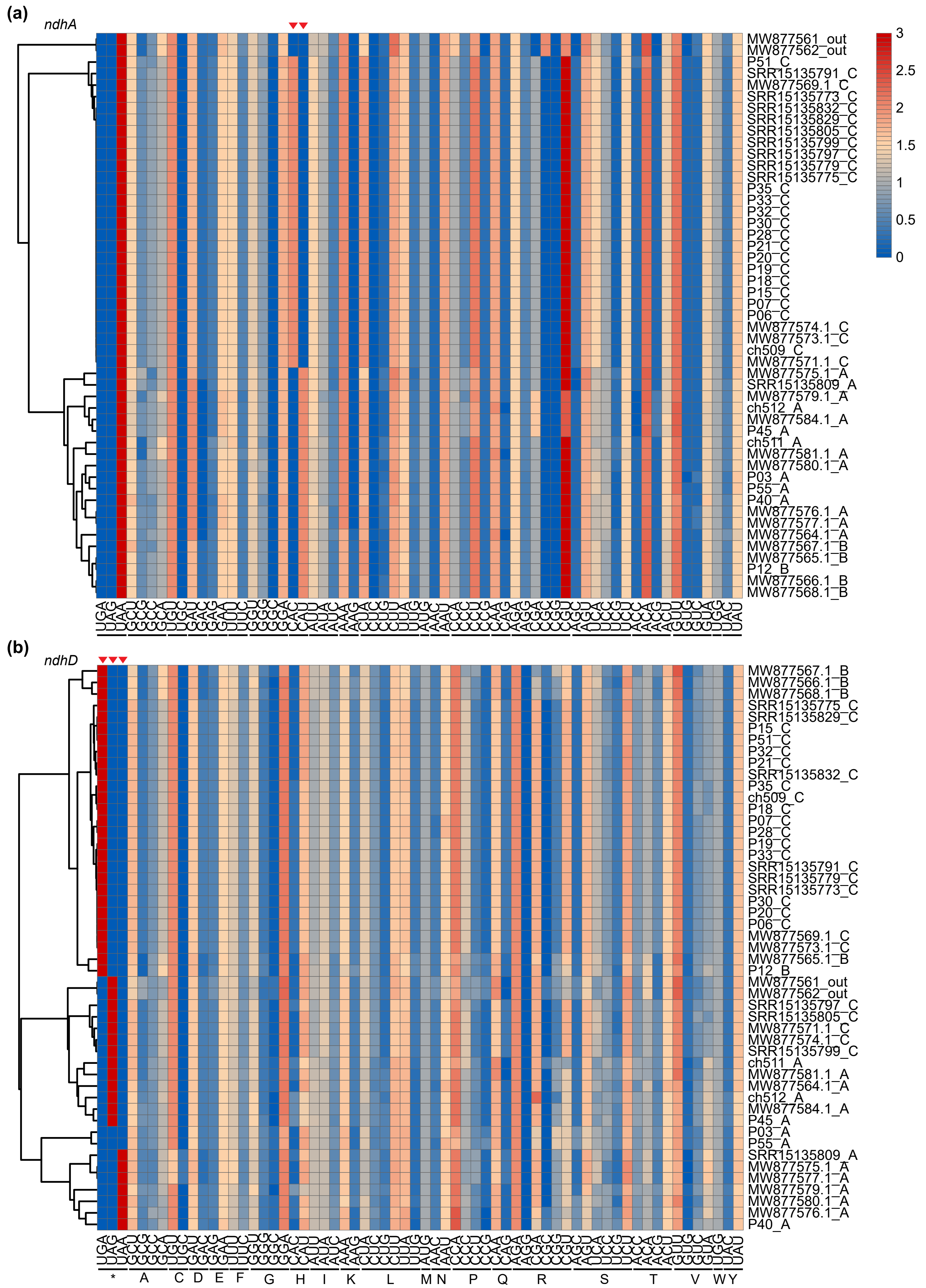
(a)
ndhA
MW877561_out
MW877562_out
P51_C
SRR15135791_C
MW877569.1_C
SRR15135773_C
SRR15135832_C
SRR15135829_C
SRR15135805_C
SRR15135799_C
SRR15135797_C
SRR15135779_C
SRR15135775_C
P35_C
P33_C
P32_C
P30_C
P28_C
P21_C
P20_C
P19_C
P18_C
P15_C
P07_C
P06_C
MW877574.1_C
MW877573.1_C
ch509_C
MW877571.1_C
MW877575.1_A
SRR15135809_A
MW877579.1_A
ch512_A
MW877584.1_A
P45_A
ch511_A
MW877581.1_A
MW877580.1_A
P03_A
P55_A
P40_A
MW877576.1_A
MW877577.1_A
MW877564.1_A
MW877567.1_B
MW877565.1_B
P12_B
MW877566.1_B
MW877568.1_B
3
2.5
2
1.5
1
0.5
0
(b)
ndhD
MW877567.1_B
MW877566.1_B
MW877568.1_B
SRR15135775_C
SRR15135829_C
P15_C
P51_C
P32_C
P21_C
SRR15135832_C
P35_C
ch509_C
P18_C
P07_C
P28_C
P19_C
P33_C
SRR15135791_C
SRR15135779_C
SRR15135773_C
P30_C
P20_C
P06_C
MW877569.1_C
MW877573.1_C
MW877565.1_B
P12_B
MW877561_out
MW877562_out
SRR15135797_C
SRR15135805_C
MW877571.1_C
MW877574.1_C
SRR15135799_C
ch511_A
MW877581.1_A
MW877564.1_A
ch512_A
MW877584.1_A
P45_A
P03_A
P55_A
SRR15135809_A
MW877575.1_A
MW877577.1_A
MW877579.1_A
MW877580.1_A
MW877576.1_A
P40_A
* A C D E F G H I K L M N P Q R S T V W Y

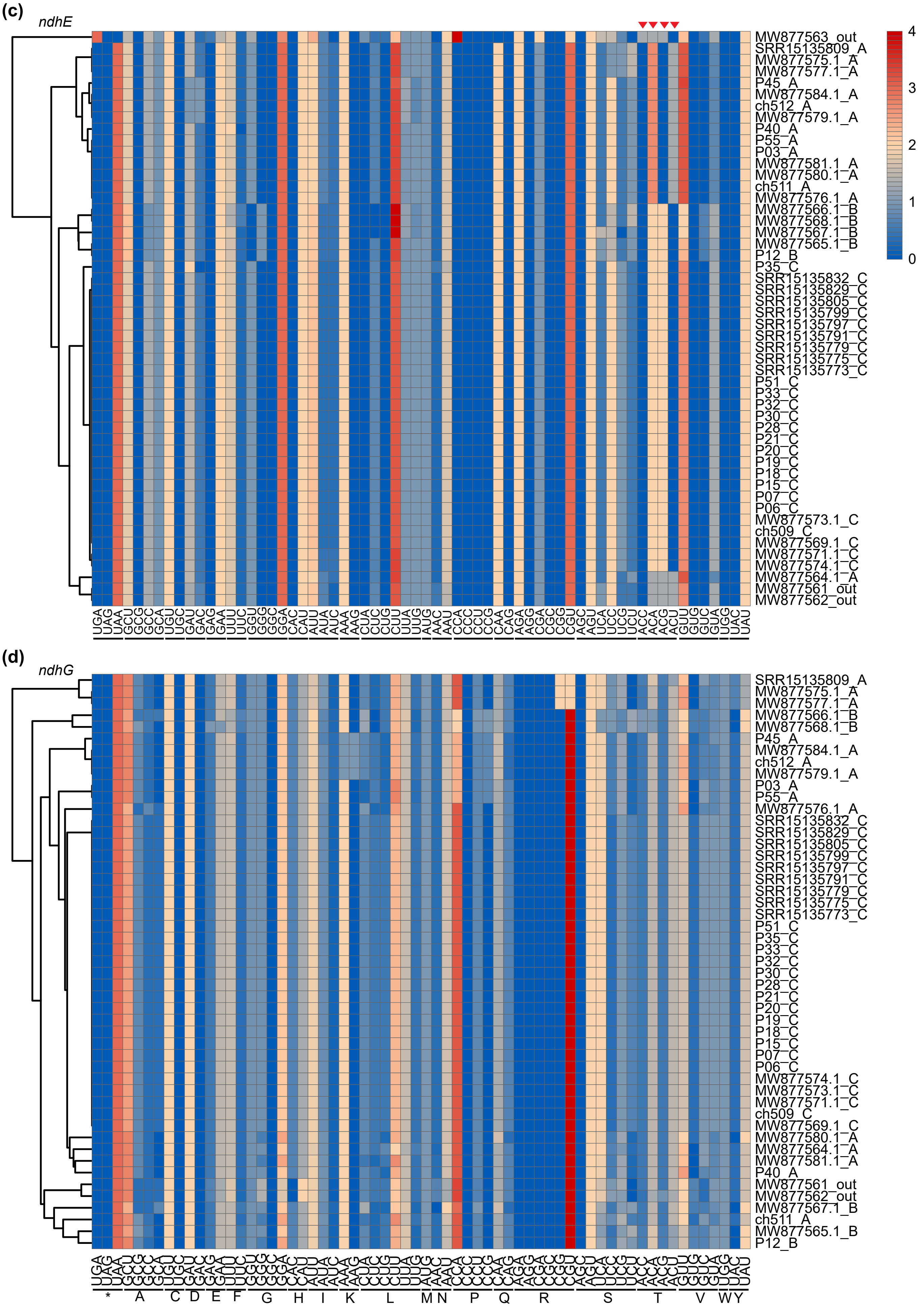

(c)
ndhE
MW877563_out
SRR15135809_A
MW877575.1_A
MW877577.1_A
P45_A
MW877584.1_A
ch512_A
MW877579.1_A
P40_A
P55_A
P03_A
MW877581.1_A
MW877580.1_A
ch511_A
MW877576.1_A
MW877566.1_B
MW877568.1_B
MW877567.1_B
MW877565.1_B
P12_B
P35_C
SRR15135832_C
SRR15135829_C
SRR15135805_C
SRR15135799_C
SRR15135797_C
SRR15135791_C
SRR15135779_C
SRR15135775_C
SRR15135773_C
P51_C
P33_C
P32_C
P30_C
P28_C
P21_C
P20_C
P19_C
P18_C
P15_C
P07_C
P06_C
MW877573.1_C
ch509_C
MW877569.1_C
MW877571.1_C
MW877574.1_C
MW877564.1_A
MW877561_out
MW877562_out
4
3
2
1
0
UGA UAG UAA GCU GCG GCC GCA UGU UGC GAU GAC GAG GAA UUU UUC GGU GGG GGC GGA CAC CAU AUU AUA AUC AAA AAG CUA CUC CUG CUU UUA UUG AUG AAC AAU CCA CCC CCU CCG CAA CAG AGA AGG CGA CGC CGG CGU AGC AGU UCA UCC UCG UCU ACC ACA ACG ACU GUU GUG GUC GUA UGG UAC UAU
(d)
ndhG
SRR15135809_A
MW877575.1_A
MW877577.1_A
MW877566.1_B
MW877568.1_B
P45_A
MW877584.1_A
ch512_A
MW877579.1_A
P03_A
P55_A
MW877576.1_A
SRR15135832_C
SRR15135829_C
SRR15135805_C
SRR15135799_C
SRR15135797_C
SRR15135791_C
SRR15135779_C
SRR15135775_C
SRR15135773_C
P51_C
P35_C
P33_C
P32_C
P30_C
P28_C
P21_C
P20_C
P19_C
P18_C
P15_C
P07_C
P06_C
MW877574.1_C
MW877573.1_C
MW877571.1_C
ch509_C
MW877569.1_C
MW877580.1_A
MW877564.1_A
MW877581.1_A
P40_A
MW877561_out
MW877562_out
MW877567.1_B
ch511_A
MW877565.1_B
P12_B
UGA UAG UAA GCU GCG GCC GCA UGU UGC GAU GAC GAG GAA UUU UUC GGU GGG GGC GGA CAC CAU AUU AUA AUC AAA AAG CUA CUC CUG CUU UUA UUG AUG AAC AAU CCA CCC CCU CCG CAA CAG AGA AGG CGA CGC CGG CGU AGC AGU UCA UCC UCG UCU ACC ACA ACG ACU GUU GUG GUC GUA UGG UAC UAU
* A C D E F G H I K L M N P Q R S T V W Y

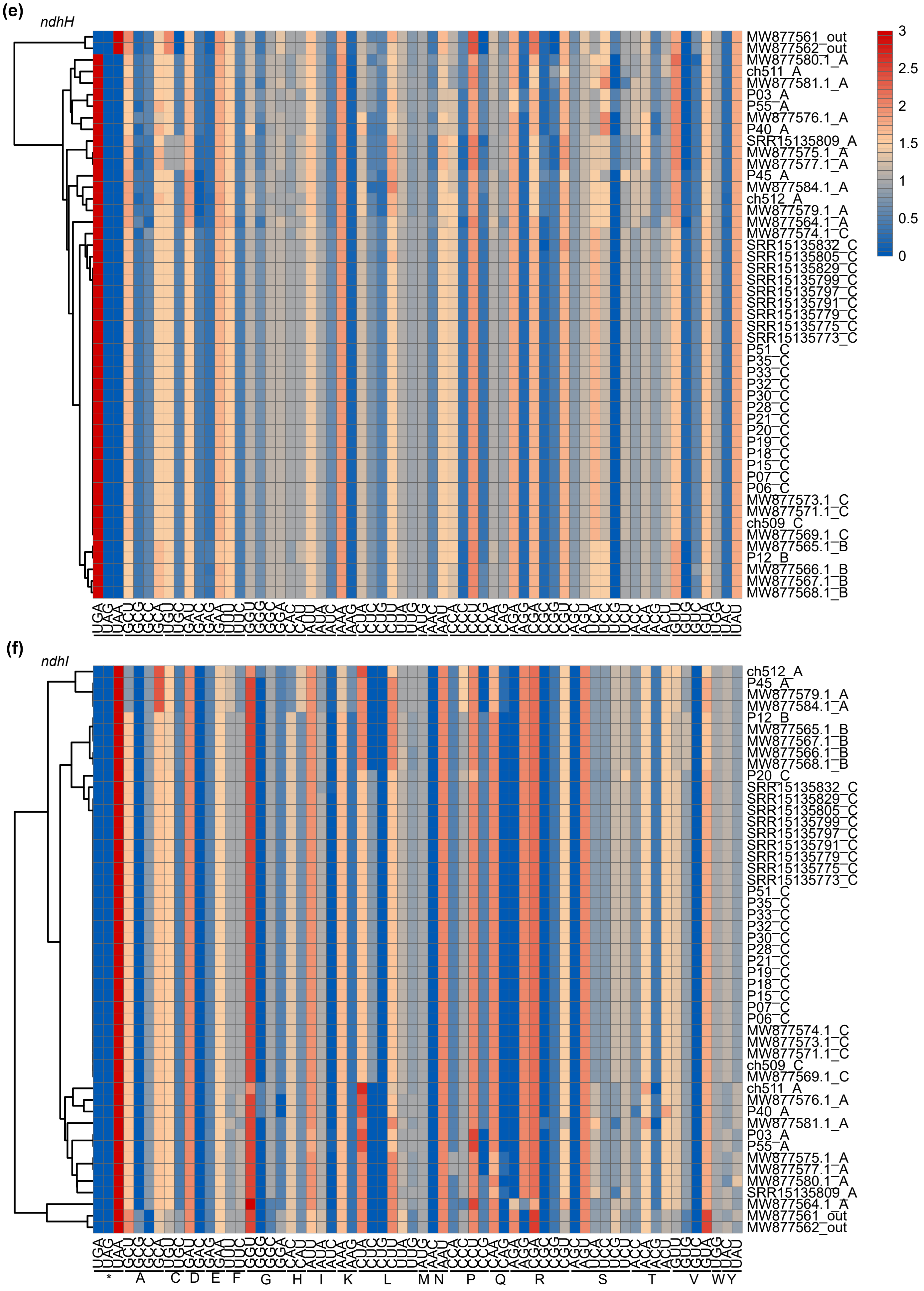
(e)
ndhH
3
2.5
2
1.5
1
0.5
0
MW877561_out
MW877562_out
MW877580.1_A
ch511_A
MW877581.1_A
P03_A
P55_A
MW877576.1_A
P40_A
SRR15135809_A
MW877575.1_A
MW877577.1_A
P45_A
MW877584.1_A
ch512_A
MW877579.1_A
MW877564.1_A
MW877574.1_C
SRR15135832_C
SRR15135805_C
SRR15135829_C
SRR15135799_C
SRR15135797_C
SRR15135791_C
SRR15135779_C
SRR15135775_C
SRR15135773_C
P51_C
P35_C
P33_C
P32_C
P30_C
P28_C
P21_C
P20_C
P19_C
P18_C
P15_C
P07_C
P06_C
MW877573.1_C
MW877571.1_C
ch509_C
MW877569.1_C
MW877565.1_B
P12_B
MW877566.1_B
MW877567.1_B
MW877568.1_B
(f)
ndhI
ch512_A
P45_A
MW877579.1_A
MW877584.1_A
P12_B
MW877565.1_B
MW877567.1_B
MW877566.1_B
MW877568.1_B
P20_C
SRR15135832_C
SRR15135829_C
SRR15135805_C
SRR15135799_C
SRR15135797_C
SRR15135791_C
SRR15135779_C
SRR15135775_C
SRR15135773_C
P51_C
P35_C
P33_C
P32_C
P30_C
P28_C
P21_C
P19_C
P18_C
P15_C
P07_C
P06_C
MW877574.1_C
MW877573.1_C
MW877571.1_C
ch509_C
MW877569.1_C
ch511_A
MW877576.1_A
P40_A
MW877581.1_A
P03_A
P55_A
MW877575.1_A
MW877577.1_A
MW877580.1_A
SRR15135809_A
MW877564.1_A
MW877561_out
MW877562_out
* A C D E F G H I K L M N P Q R S T V W Y

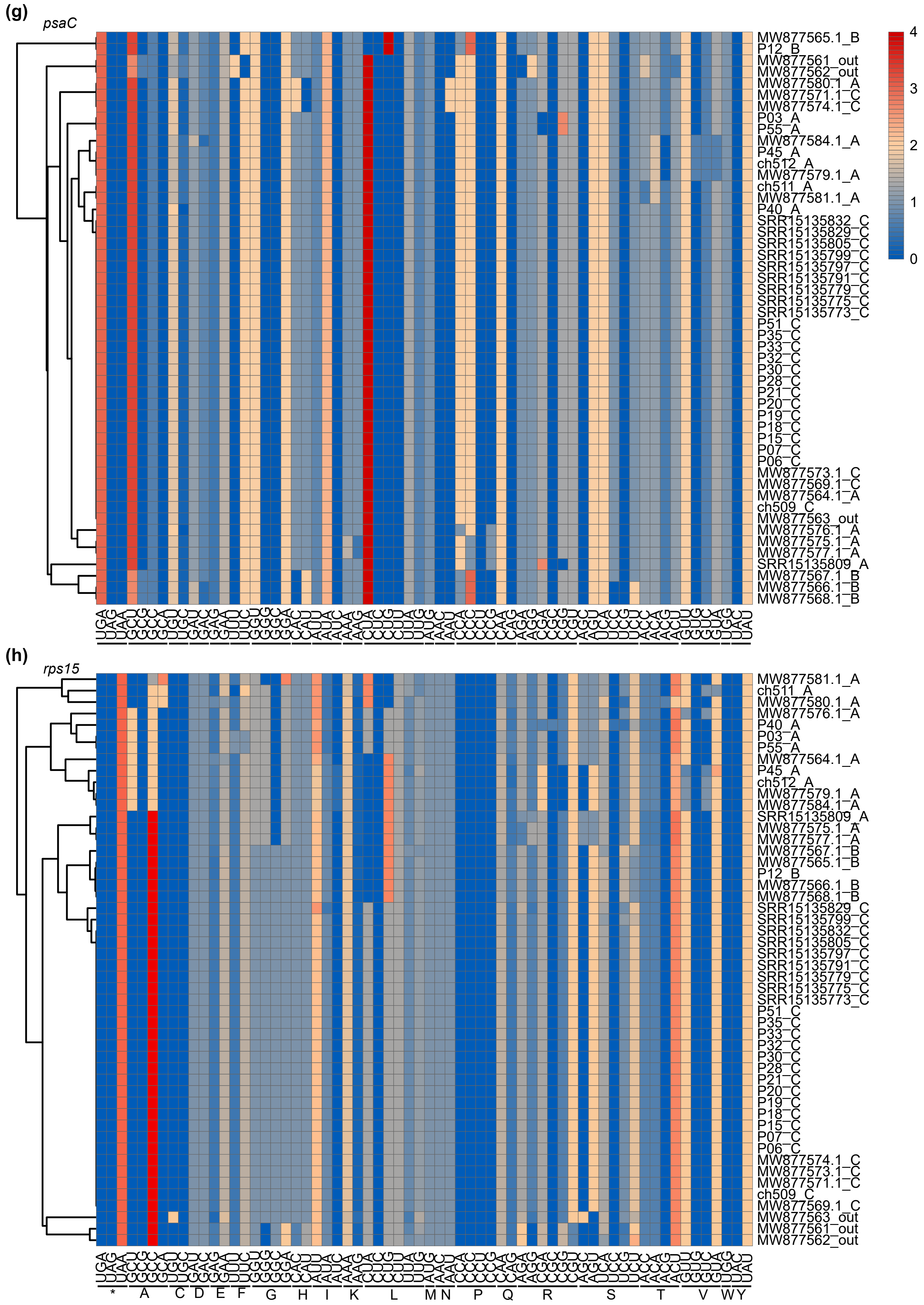

(g)
psaC
4
3
2
1
0
MW877565.1_B
P12_B
MW877561_out
MW877562_out
MW877580.1_A
MW877571.1_C
MW877574.1_C
P03_A
P55_A
MW877584.1_A
P45_A
ch512_A
MW877579.1_A
ch511_A
MW877581.1_A
P40_A
SRR15135832_C
SRR15135829_C
SRR15135805_C
SRR15135799_C
SRR15135797_C
SRR15135791_C
SRR15135779_C
SRR15135775_C
SRR15135773_C
P51_C
P35_C
P33_C
P32_C
P30_C
P28_C
P21_C
P20_C
P19_C
P18_C
P15_C
P07_C
P06_C
MW877573.1_C
MW877569.1_C
MW877564.1_A
ch509_C
MW877563_out
MW877576.1_A
MW877575.1_A
MW877577.1_A
SRR15135809_A
MW877567.1_B
MW877566.1_B
MW877568.1_B
(h)
rps15
MW877581.1_A
ch511_A
MW877580.1_A
MW877576.1_A
P40_A
P03_A
P55_A
MW877564.1_A
P45_A
ch512_A
MW877579.1_A
MW877584.1_A
SRR15135809_A
MW877575.1_A
MW877577.1_A
MW877567.1_B
MW877565.1_B
P12_B
MW877566.1_B
MW877568.1_B
SRR15135829_C
SRR15135799_C
SRR15135832_C
SRR15135805_C
SRR15135797_C
SRR15135791_C
SRR15135779_C
SRR15135775_C
SRR15135773_C
P51_C
P35_C
P33_C
P32_C
P30_C
P28_C
P21_C
P20_C
P19_C
P18_C
P15_C
P07_C
P06_C
MW877574.1_C
MW877573.1_C
MW877571.1_C
ch509_C
MW877569.1_C
MW877563_out
MW877561_out
MW877562_out
* A C D E F G H I K L M N P Q R S T V W Y

(i)

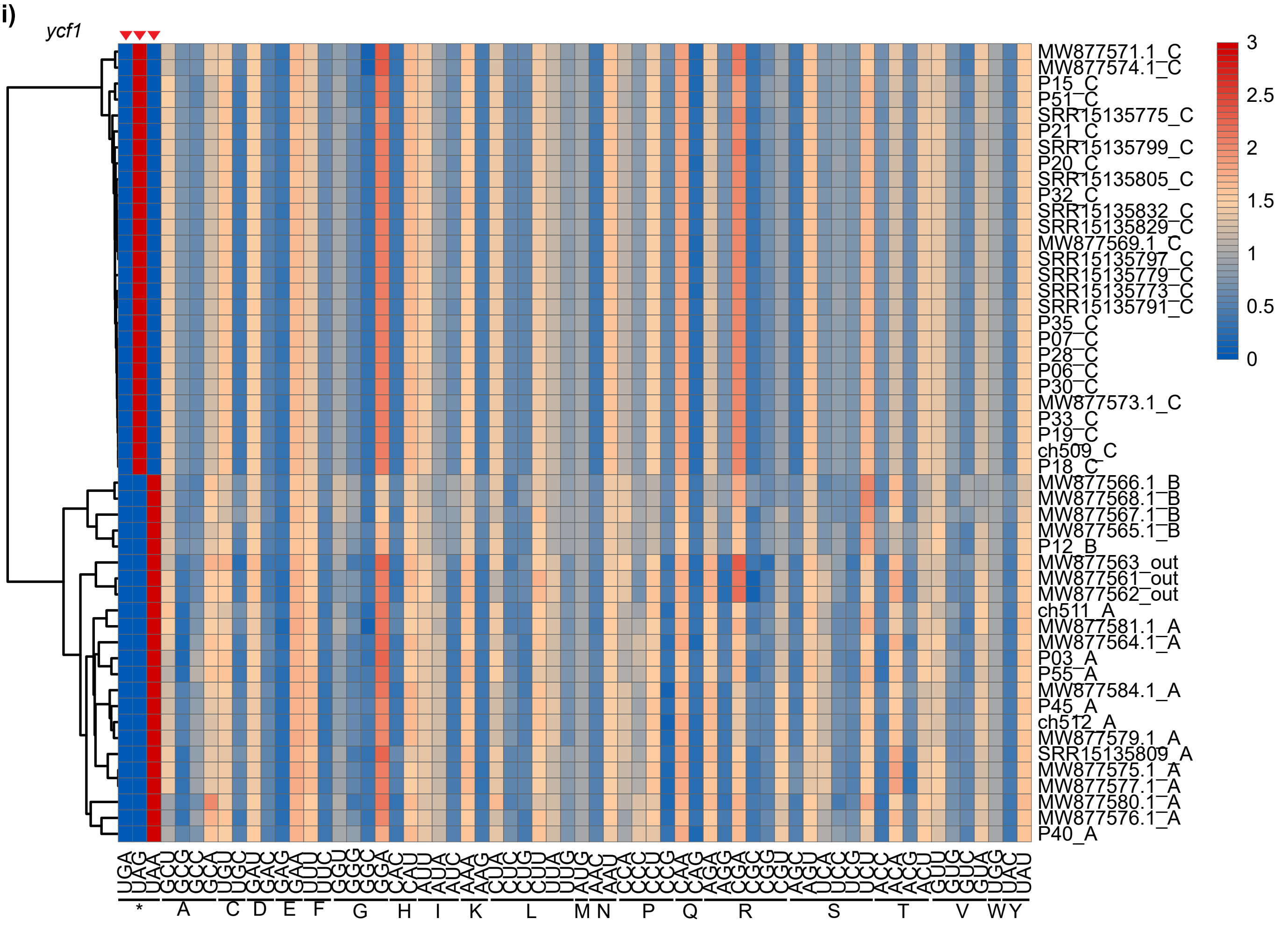

Fig. S10 Relative synonymous codon usage (RSCU) of relocated genes. (a) *ndhA*; **(b)** *ndhD*; **(c)** *ndhE*; **(d)** *ndhG*; **(e)** *ndhH*; **(f)** *ndhI*; **(g)** *psaC*; **(h)** *rps15*; and **(i)** *ycf1*. Clades on the left indicate the clusters based on RSCU of each CDS. Red triangles on the top mark serious divergences.

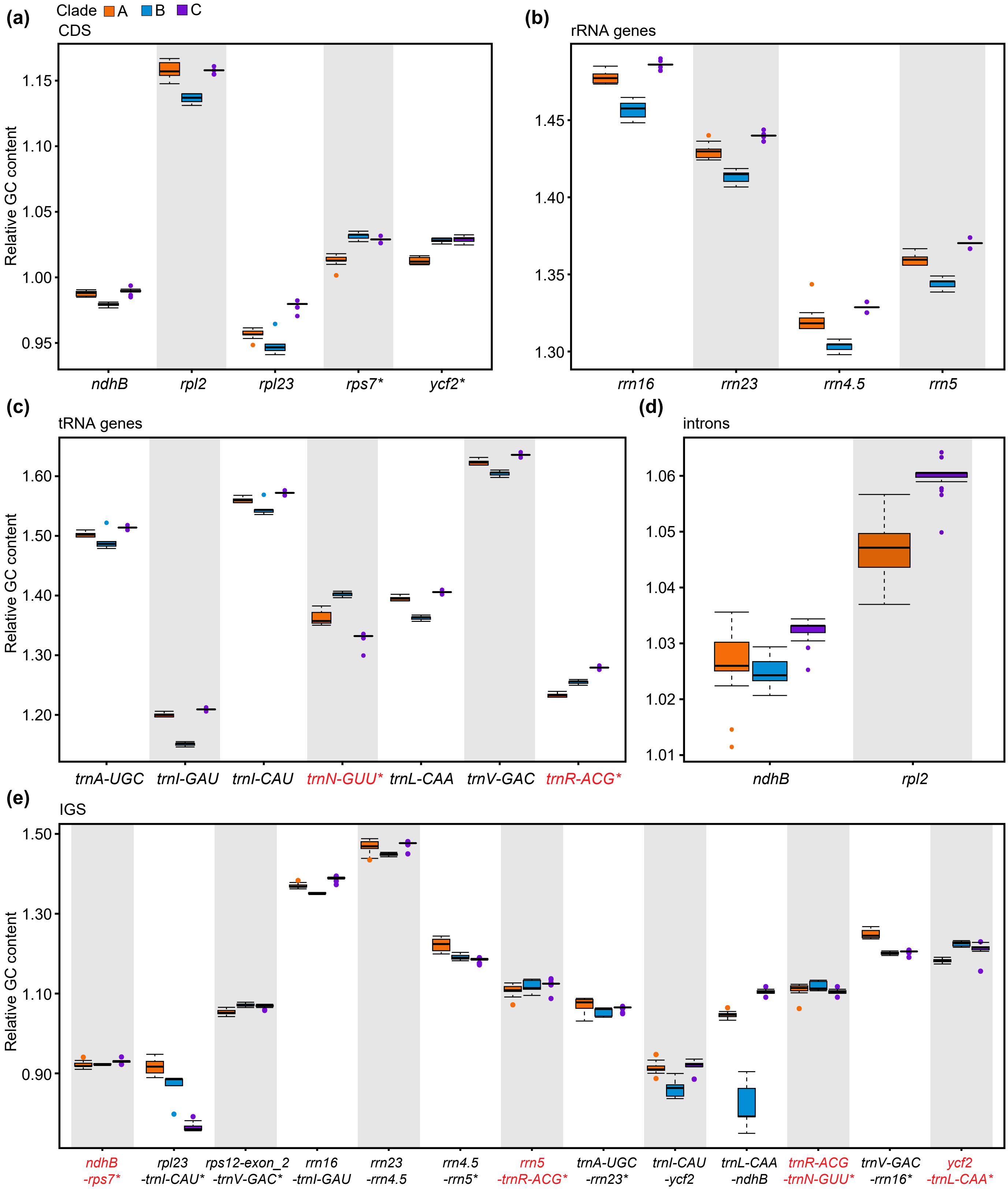

Fig. S11 Changes of relative GC content (divided by total GC content) of CIR loci. Relative GC content of non-relocated **(a)** coding sequences (CDS); **(b)** ribosomal RNA (rRNA) genes; **(c)** transfer RNA (tRNA) genes; **(d)** introns; and **(e)** intergenic spacers (IGS). Loci with asterisk denote a decline in Clade C and the loci close to inversion breaking point are marked in red font.

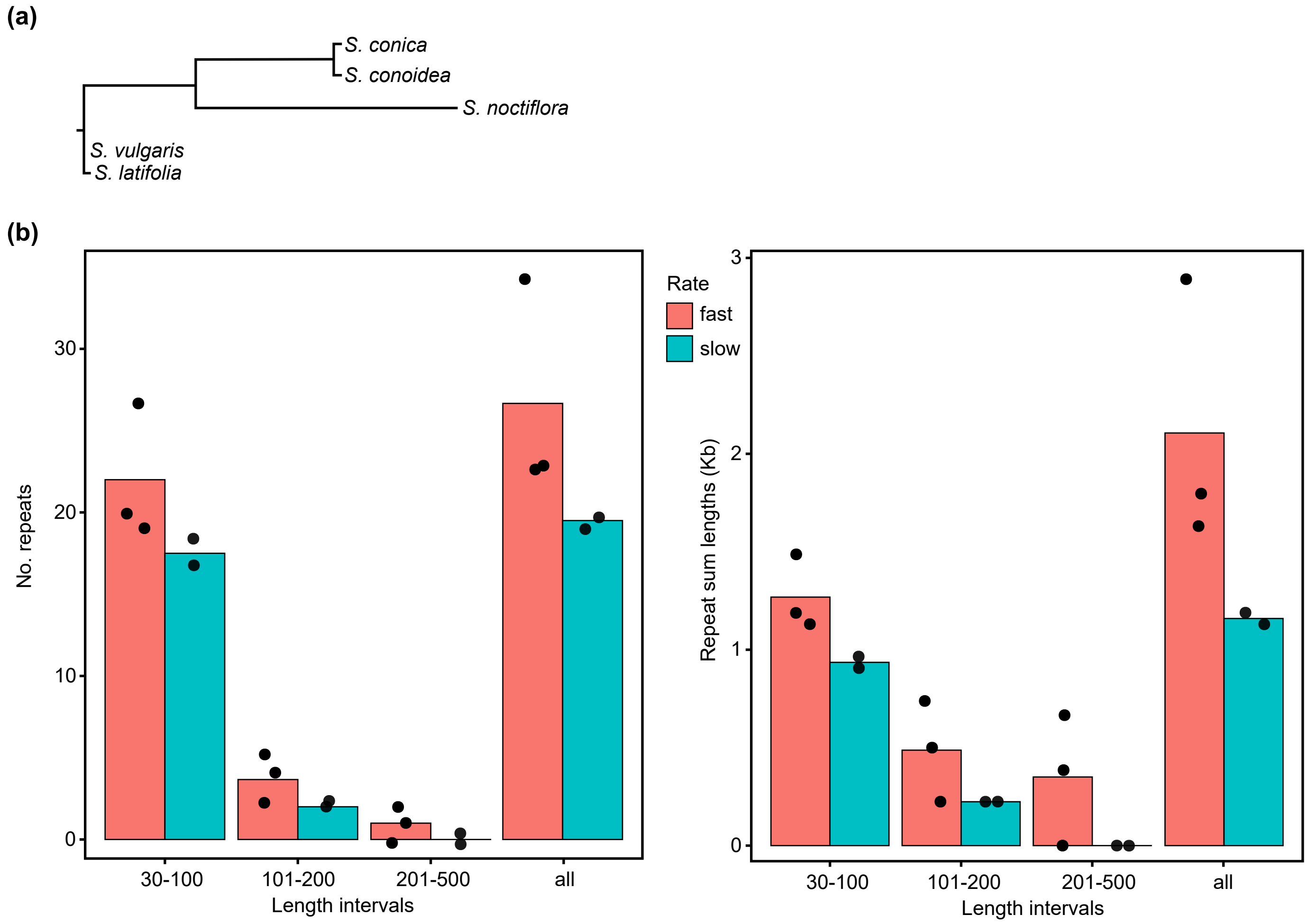

Fig. S12 Plastid repeats in *Silene* species with different evolutionary rates cited from Abdel Ghany et al. (2022). **(a)** Evolutionary rates across five *Silene* species. **(b)** Sum counts and lengths of repeats in species with fast (red) and slow (blue) rates.
